# Systematic investigation on quad-metallic AgAuPdPt and tri-metallic AuPdPt NPs through the solid-state dewetting of quad-layer Ag/Au/Pd/Pt thin films on c-plane sapphire

**DOI:** 10.1371/journal.pone.0224208

**Published:** 2019-10-21

**Authors:** Mao Sui, Sundar Kunwar, Puran Pandey, Sanchaya Pandit, Jihoon Lee

**Affiliations:** 1 Institute of Hybrid Materials, College of Materials Science and Engineering, Qingdao University, Qingdao, P. R. China; 2 Department of Electronic Engineering, College of Electronics and Information, Kwangwoon University, Nowon-gu Seoul, South Korea; Institute of Materials Science, GERMANY

## Abstract

Multi-metallic alloy nanoparticles (MNPs) can offer valuable opportunities to meet the various demands of applications. MNPs consist of various noble metallic elements can combine diverse electronic, optical and catalytic properties in a single NP configuration, thus taking the advantage of each element. In this paper, the fabrication of tri- and quad- metallic alloy NPs with noble elements (Ag, Au, Pd and Pt) and the corresponding localized surface plasmon resonance (LPSR) properties are systematically demonstrated. Tri- and quad-metallic alloy NPs come in various size and configurations by the solid-state dewetting of Ag/Au/Pd/Pt quad-layers on sapphire (0001). Tri-metallic AuPdPt NPs are demonstrated by the systematic control of growth temperature along with the significant Ag atom sublimation. Strongly enhanced and tunable LPSR is exerted in the UV-VIS regions depending upon the size, configuration, spacing and elemental composition of the MNPs. The size dependent LSPR response of MNPs is discussed based on the absorption and scattering along with the excitation of dipolar, quadrupolar, high order and multipolar resonance modes. The MNPs exhibit much stronger and dynamic LSPR bands as compared with the monometallic Pt and Pd NPs with the comparable size and configurations.

## Introduction

The localized surface plasmon resonance (LSPR) offered by the noble metallic NPs has been an intensive area of research and thus utilized in various fields to increase absorption and scattering of selective wavelength, which can effectively enhance the energy conversion efficiency in solar cells, light scattering in LEDs, photocatalytic activity in fuel cells and sensitivity in the biological sensors [1–13]. Multi-metallic alloy nanoparticles (MNPs) are important materials systems in various fields due to the feasibility of integrating beneficial electronic, optical and catalytic properties of each element [1,2,6,7]. The properties and functionalities of MNPs are largely dependent on the elemental composition along with the morphology [8–12]. As an example, the tri-metallic PdAgCd NPs, in contrast to the mono-metallic Pd and bi-metallic PdAg NPs, demonstrated a significantly enhanced hydrogen storage efficiency due to the synergetic effect of alloy elements [14]. Among various noble metallic elements, the Au and Ag are widely adapted as superior plasmonic materials due to their strong generation of electromagnetic fields at the resonance frequency. Meanwhile, the Pd and Pt NPs can offer superior catalytic activity and stability and thus are promising cathode materials in the fuel cells [15,16]. The combination of these elements can deliver interesting opportunities, i.e. a superior photo-catalytic system for the fuel cells, by taking the advantage of each element. At the same time, the solid-state dewetting (SSD) can offer an efficient route to fabricate the MNPs based on the temperature-induced surface diffusion and intermixing process below the melting point of elements [17–20]. Various mono- and bi-metallic NPs have been successfully applied in numerous applications based on the SSD, in which the initial layer thickness, growth conditions and substrate properties can largely affect the resulting crystal orientation, size and shape of NPs [21]. Meanwhile, the MNPs have been generally synthesized in the form of colloidal solution by the chemical approaches due to the relative handy synthesis environment, which can provide precise control on size, shape and uniformity [22]. However, the device performance with the chemically synthesized NPs has been somewhat hindered by the issues of impurity, durability and weak binding to substrate. The SSD approach can provide highly stable, pure and large scale-fabrication of various monometallic and multi-metallic alloy NPs [23]. The detailed study on the fabrication and characterizations of MNPs by the SSD can allow one to control the composition and morphology of NPs for the target applications, which however has not been explored for the high order MNPs up to now.

In this paper, a systematic study on the fabrication and LSPR property of MNPs is performed by means of the solid-state dewetting (SSD) of Ag/Au/Pd/Pt quad-metallic layers on sapphire (0001). A wide range of size, density and configuration of quad-metallic AgAuPdPt and tri-metallic AuPdPt NPs have been successfully demonstrated depending on the control of initial quad-layer thickness and growth conditions, which exhibits strong and dynamic LSPR bands in the UV and VIS wavelengths. The fabrication and evolution of various MNPs are discussed based on the temperature-dependent atomic inter-diffusion, alloy phase, energy minimization and sublimation of Ag atoms along with the systematic analyses. The LSPR bands demonstrate the dynamic variations in the intensity, position and bandwidth based on the surface morphology and composition of MNPs.

## Materials and methods

In this work, the double-side polished 430 μm-thick c-plane sapphire with ± 0.1° off-axis (iNexus, South Korea) was chosen as a substrate. Firstly, the sapphire (0001) substrates were treated with a thermal degassing in a pulsed laser deposition (PLD) chamber (DaDa TG, South Korea) at 600 °C for 30 min under 1 × 10^−4^ Torr to remove the surface contaminants such as vapors and trapped gases. The surface morphology and optical properties of degassed bare sapphire (0001) is provided in S1 Fig. Then, the sequent deposition of Ag/Au/Pd/Pt quad-layer films on sapphire (0001) performed by means of sputtering in an ion-coater (COXEM, South Korea) under vacuum (1 × 10^−1^ Torr). All the metal layers were deposited at constant growth rate of 0.05 nm/s with 3 mA ionization current. The surface morphology of as-deposited Ag_0.46_Au_0.18_Pd_0.18_Pt_0.18_ quad-layer samples with a thickness between 3.4 and 85 nm is provided in S2 Fig. For the fabrication of various configurations of multi-metallic alloy nanostructures, the multilayer film thickness, composition and annealing temperature (Ta) were systematically varied. In the 1^st^ set, the total thickness was varied between 3.4 and 85 nm for a fixed thickness composition of Ag_0.46_Au_0.18_Pd_0.18_Pt_0.18_ (Ag_0.46_ denotes that the Ag thickness was 46% of total thickness). In the 2^nd^ and 3^rd^ sets, the multilayer thickness was fixed such as Ag_8 nm_ / Au_3 nm_ / Pd_3 nm_ / Pt_3 nm_ and Ag_24 nm_ / Au_9 nm_ / Pd_9 nm_ / Pt_9 nm_ and annealed at different temperatures between 500 and 900 °C for 120 s. The higher diffusivity metallic element, i.e. Ag, was firstly deposited in order to improve the dewetting degree. Once ready, each sample was annealed at the pre-determined target Ta under 1 × 10^−4^ Torr in a PLD chamber. The 1^st^ thickness variation set was annealed at a fixed temperature of 850 °C while the other two sets were annealed between 500 and 900 °C. The target temperature was reached by the ramping rate of 4 °C/s and dwelt for 120 s for the NP maturation.

Once finished each growth, the morphological and elemental analyses of multi-metallic NPs were performed by an atomic force microscope (AFM) (XE-70, Park Systems Corp., South Korea), scanning electron microscope (SEM) (CX-200, COXEM, South Korea and Regulus 8230, Hitachi, Japan) and energy-dispersive x-ray spectroscope (EDS) (Noran System 7, Thermo Fisher, United States of America and Ultimax, Oxford Instruments, United Kingdom). The AFM scanning was performed in a non-contact mode under an ambient using the same batch of AFM tips to maintain the consistency and minimized the tip effects on the NPs morphology. The optical properties of multi-metallic NPs were characterized by a NOST system (Nostoptiks, South Korea) equipped with the ANDOR spectrograph sr500i, CCD detector and UV-VIS-NIR light sources. The reflectance and transmittance spectra were experimentally measured by the normal incidence of light and the extinction were calculated by extinction (%) = 100%—(reflectance + transmittance) %. In addition, to visualize the local E-field distribution of multi-metallic NPs, the finite difference time domain (FDTD) simulation (Lumerical Solutions, Canada) was performed with the dielectric constants taken from the Palik’s and Rakic’s models [24,25]. Real NPs from the AFM images were imported into the simulations and the dielectric constants for specific alloy compositions was averaged from the dielectric constant of pure Au, Pd and Pt based on the at. % fraction [20,26]. The total field scattered field (TFSF) wave source of 250 to 1000 nm wavelength was engaged from the z-direction to excite the NP on sapphire. The perfectly matched layer (PML) boundary condition was applied in all direction. To avoid the interference effects, the gap between the PML boundary and nanostructure was about 500 nm in all direction. The auto shut-off level of 10^−6^ and 3D mesh grid of 0.5 to 5 nm were used.

## Results and discussion

**Fig 1:**
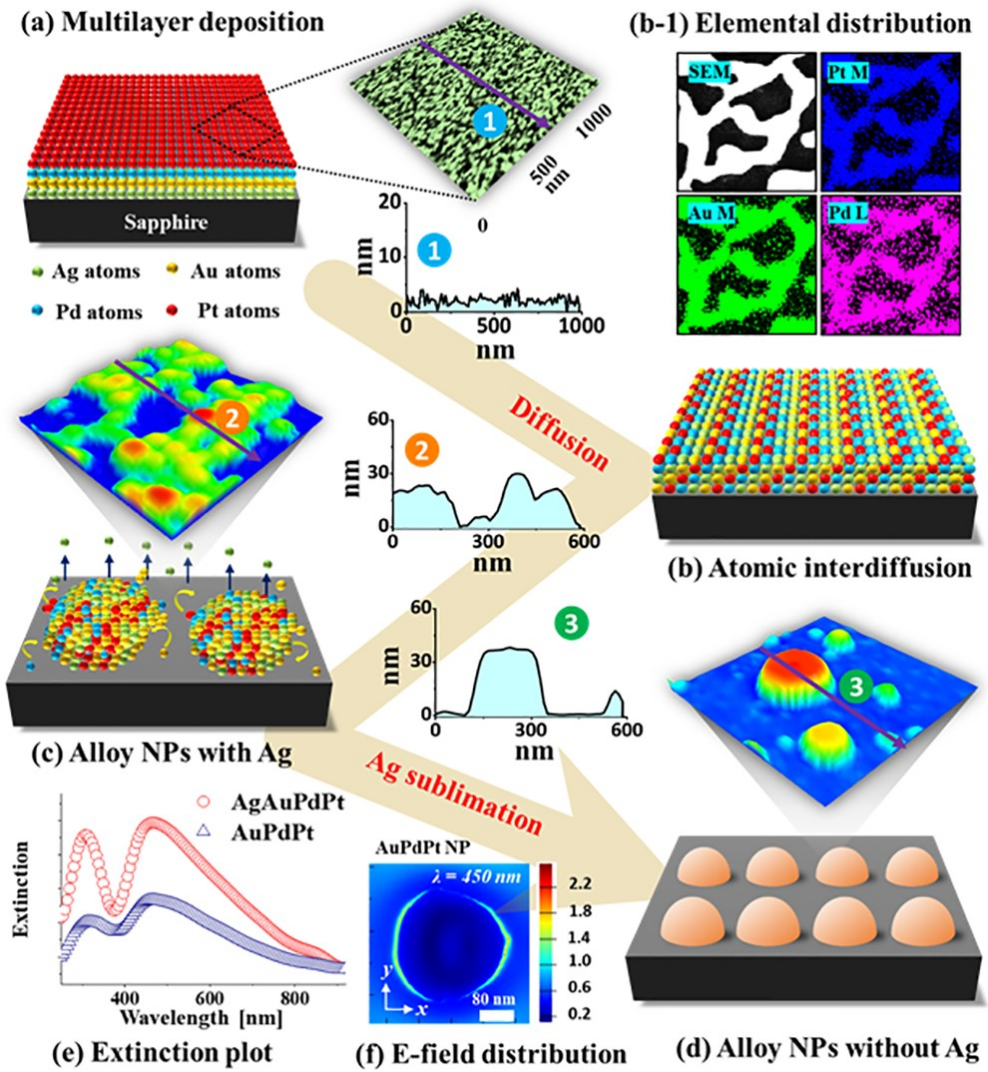
Illustration of the fabrication process of multimetallic alloy nanoparticles (MNPs) on sapphire (0001) by the annealing of Ag/Au/Pd/Pt quad-layer thin films. (a) Deposition schematic of Ag/Au/Pd/Pt quad-layers. The inset shows an atomic force microscope (AFM) image and a cross-sectional line profile of as-deposited film. (b)–(b-1) Atomic interdiffusion and elemental distribution after annealing. (c) Formation of MNPs. The typical AFM images of quad-metallic NPs and line profile are shown in insets. (d) Evolution of tri-metallic AuPdPt alloy NPs after Ag sublimation. (e) Extinction plot of AgAuPdPt and AuPdPt alloy NPs. (f) E-field distribution of the AuPdPt alloy NP.

Fig 1 shows the overall fabrication process of multi-metallic alloy NPs (MNPs) of Ag, Au, Pd and Pt by the systematic dewetting of Ag/Au/Pd/Pt quad-layers of various thickness. The quad-layer films were sequentially deposited as shown in Fig 1(a) and then annealed under vacuum below the melting point of constituent metallic elements. The average surface height and roughness of as-deposited multilayer film was less than 5 nm in S2 Fig. Since the annealing temperature (Ta) was lower than the melting point of all elements utilized, the formation of MNPs can be expected to occur based on the solid-state dewetting (SSD) of the quad-layer films. The SSD of thin films directly depends upon the atomic diffusivity, surface energy, interface energy, initial film thickness and annealing conditions [21,27,28]. In the case of multi-metallic films, additional parameters such as the interdiffusion, alloying and interface energies between different metal layers may also affect the SSD process. Nevertheless, the SSD of thin films can be governed by the atomic diffusion and driven by the energy minimization of the thermodynamic system. In general, the Au-Pt and Ag-Pt are immiscible in the bulk phase whereas the Ag-Au and Pd-Pt are completely miscible in all compositions [29,30]. However, due to the nanoscale nature of thin films with numerous atomic vacancies and defects, the Ag, Au, Pd and Pt atoms can be well-miscible upon annealing under vacuum. Generally, the sputtered film can consists of number of atomic vacancies, native defects and grain boundaries due to the limited diffusion of atoms. During the deposition of multiple metal layers, the metal atoms can enter in the existing vacany of the underlying layer to form partially intermixed interface, which can be further intermixed upon annealing due to the interdiffusion of atoms. In the Ag/Au/Pd/Pt configuration, the highly diffusive atoms, i.e. Ag or Au, in the bottom layers can be activated first and start to interdiffuse through the interfaces. For instance, the interface energy of Pt atoms on sapphire is high while the surface diffusitivity is low as compared to that of Ag and Au aotms. Thus, the deposition sequence also play crucial role on the dewetting process of multilayer films. The samples was annealed from the substrate side and thus the heat transfer could be from the bottom to the top-layers [28]. Thus, the high diffusivity metal atoms should be activated first and interdiffuse through the atomic vacancies at different metal interfaces such as Ag/Au, Au/Pd and Pd/Pt. This subsequently can result in the fully intermixed or alloyed layer that eventually nucleates and forms alloy nanostructures as displayed in Fig 1(b) and 1(c). At the same time, since the vapor pressure of Ag is very high around the temperature utilized as compared to other elements, i.e. 3 × 10^−7^ Torr at 600 °C and ~ 4.50 × 10^−5^ Torr at 850 °C, it can fully sublimate while other metallic atoms can further agglomerate to form the compact and isolated NPs as shown in Fig 1(d). Therefore, the evolution of alloy NPs can be simultaneously affected by the atomic agglomeration and sublimation of Ag atoms. As will be shown in the EDS section, the Ag atoms are completely sublimated in this set after the annealing at 850 °C and thus, the resulting nanostructures are indeed the tri-metallic AuPdPt NPs with much-enhanced diffusion due to the presence of Ag adatoms. The corresponding optical response of the alloy NPs with and without Ag varied largely as shown in Fig 1(e) and the surface morphology also had a strong influence in the LSPR peaks. For the fabrication of various shape, size and uniformity of tri- and quad-metallic alloy NPs various multilayer configuration were prepared and systematically demonstrated in the following sections.

**Fig 2:**
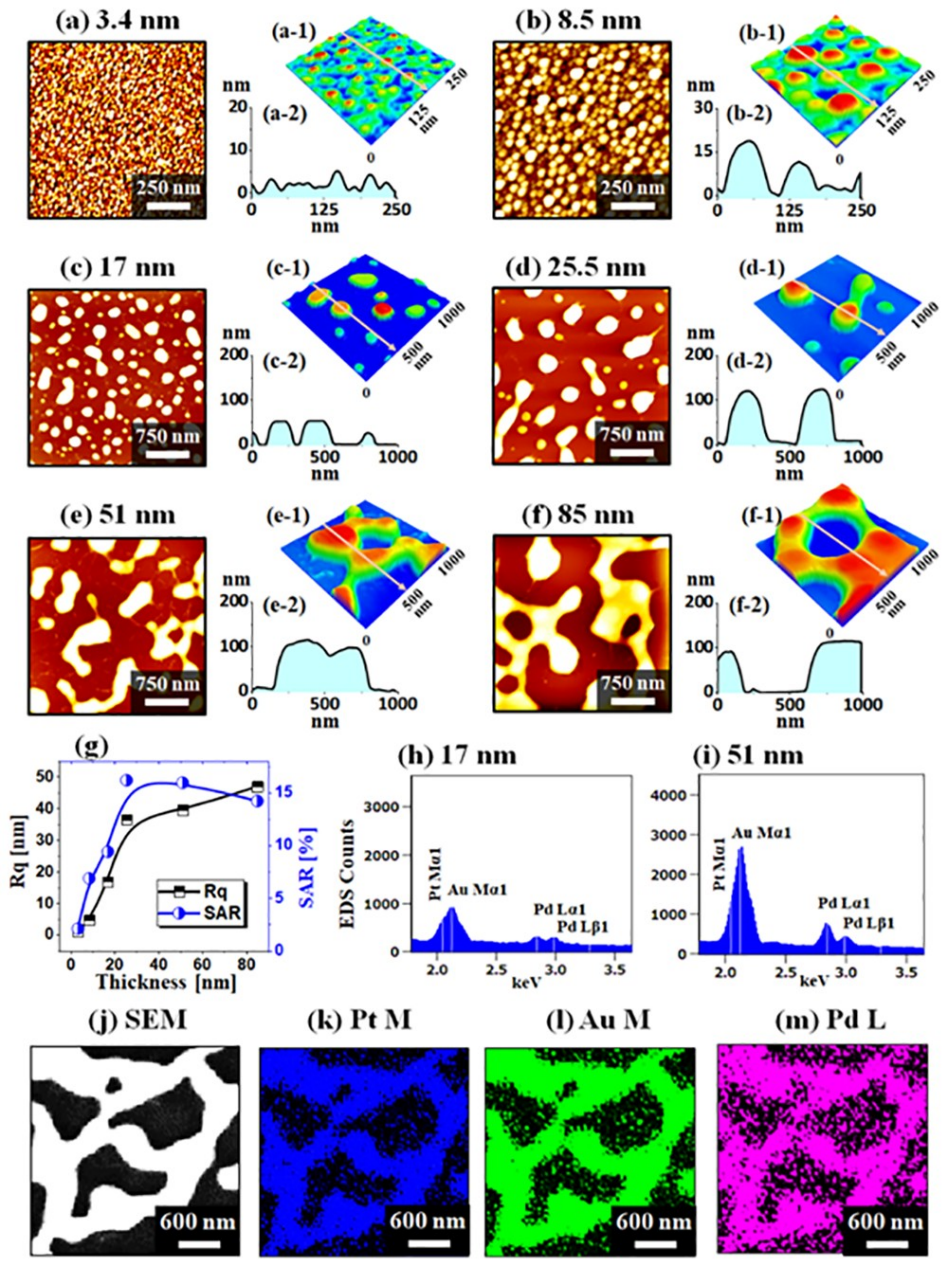
Fabrication of various AuPdPt alloy nanostructures on sapphire (0001) by the annealing of Ag/Au/Pd/Pt quad-layer thickness between 3.4 and 85 nm at fixed temperature 850 °C for 120 s. The composition of multi-layer films was Ag_0.46_Au_0.18_Pd_0.18_Pt_0.18_. (a)–(f) AFM top-views. (a-1)–(f-1) Magnified AFM side-views. (a-2)–(f-2) Cross-sectional line-profiles. (g) Summary plots of RMS roughness (Rq) and surface area ratio (SAR). (h)–(i) EDS spectra of alloy NPs with the 17 and 51 nm quad-layer thickness. (j)–(k) SEM image and Pt M, Au M, Pd L maps of alloy clusters fabricated with the 85 nm quad-layer.

The fabrication of various AuPdPt alloy nanostructures is shown in Fig 2 with the total thickness of quad-layers between 3.4 and 85 nm and a fixed compositon ratio of Ag_0.46_Au_0.18_Pd_0.18_Pt_0.18_. In this set, the annealing temperature (Ta) of 850 °C for 120 s was constant for all samples to induce sufficient diffusion of atoms and Ag sublimation. With the 3.4 nm total thickness of quad-layers, the densely packed tri-metallic AuPdPt NPs of ~ 5 nm height and 50 nm diameter were obtained as in Fig 2(a)–2(a-2). The dimensions and configuration of NPs can be clearly seen in the corresponding 3D-side views and cross-sectional line profiles. As the quad-layer film was very thin, 3.4 nm, annealing at 850 °C resulted in the small and highly dense NPs. At the same time, the NPs can instantly become thermodynamically stable upon annealing due to the favorable diffusion with the relatively smaller number of atoms [31,32]. As the thickness of quad-layers was increased to 8.5, 17, 25.5, 51 and 85 nm, the size of NPs was gradually increased due to the gradually increased absorption by the increased NP boundary while the areal density was decreased as displayed in Fig 2(b)–2(f). In specific, the spherical alloy NPs with increased spacing were formed between 3.4 and 8.5 nm and then NPs started to show an elongation up to 25.5 nm. With the additional thickness of quad-layers, the NPs can have more diffusing atoms to be absorbed and thus can result in the much larger size and at the same time, the NPs can start to coalesce in order to minimize the surface energy, resulting in the evolution of elongated and large surface coverage. The average height and diameter of tri-metallic NPs were increased from 20 to 120 nm and 100 to 300 nm between 8.5 to 25.5 nm thickness variation. With the thicker quad-layer films, the average height was mildly increased while the lateral size was expanded up to ~ 1000 nm as seen in Fig 2(e) and 2(f). The morphological evolution of various tri-metallic nanostructures can be further discussed by the RMS roughness (Rq) and surface area ratio (SAR) as shown in Fig 2(g). The Rq was sharply increased for the thickness variation between 3.4 and 25.5 nm due to the large increase in the average NP height and then mildly increased with 25.5 to 50 nm thick layers as discussed. Similarly, the SAR was also sharply increased because the surface area exerted by the tri-metallic NPs was sharply increased between 3.4 and 25.5 nm. However, the SAR was gradually reduced from the 25.5 nm along with the large degree of agglomeration of NPs. Fig 2(h)–2(m) show the elemental analyses on the tri-metallic NPs and specific EDS spectra of each sample are presented in S3 Fig. In general, no Ag peaks were observed from the EDS spectra of all samples in this series while the Au, Pd and Pt peaks were present as in Fig 2(h) and 2(i), indicating the complete sublimation of Ag atoms as discussed above. Also, the SEM image and phase maps of Pt, Au and Pd were well matched in Fig 2(j)–2(m), indicating the homogeneous distribution of Pt, Au and Pd atoms in the tri-metallic nanostructures.

**Fig 3:**
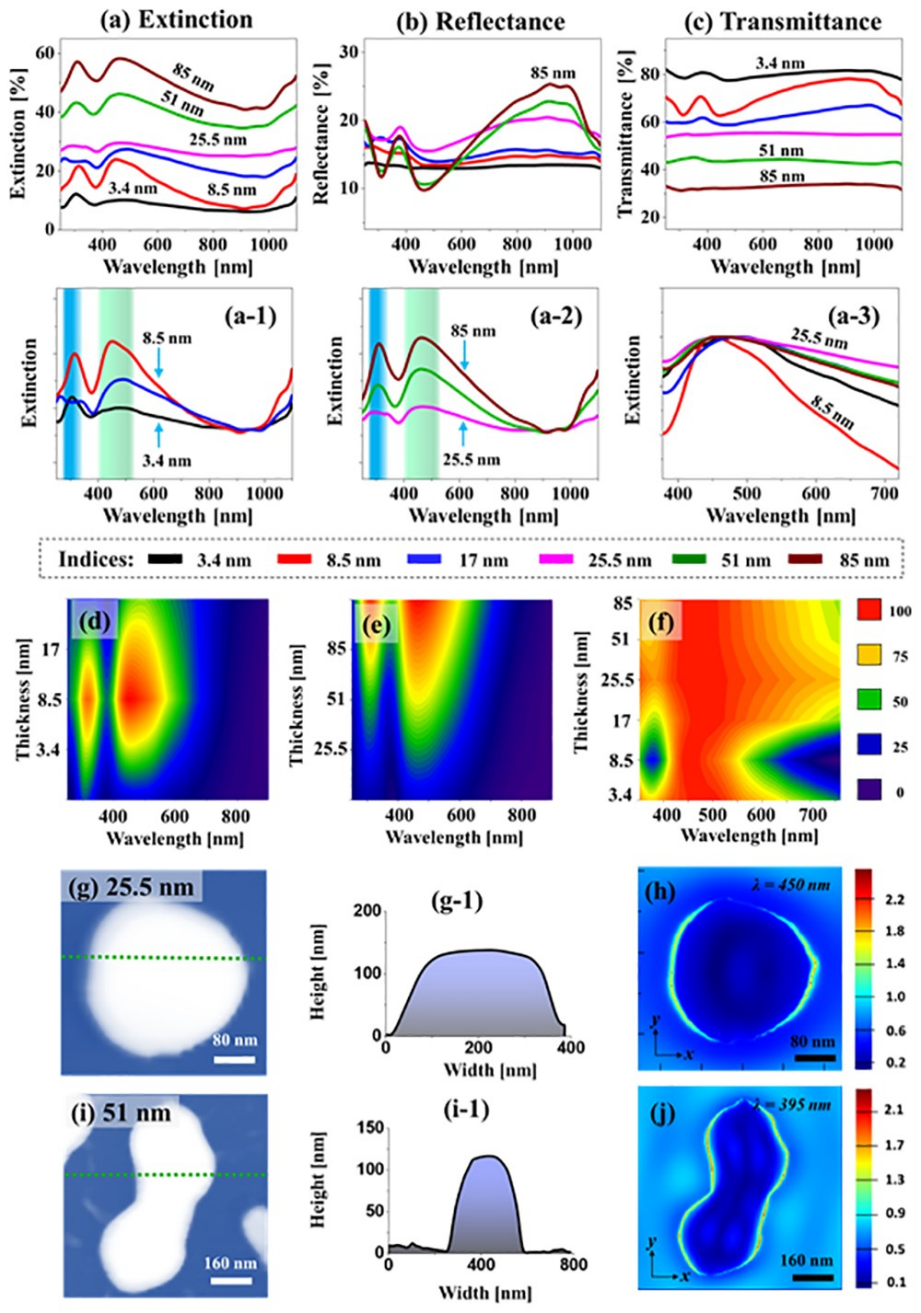
Optical properties of AuPdPt alloy nanostructures fabricated with the Ag/Au/Pd/Pt quad-layers at 850 °C for 120 s. (a)–(c) Extinction, reflectance and transmittance spectra. (a-1)–(a-3) Normalized extintion spectra. (d)–(f) Contour maps of the extinction spectra in (a-1)–(a-3). (g) and (i) AFM images of the sperical and elongated AuPdPt NPs selected for finite difference time domain (FDTD) simulations. (g-1) and (i-1) Cross-sectional line profiles. (h) and (j) E-field distribution at resonance wavelengths in xy-plane.

Fig 3 shows the LSPR properties of the tri-metallic AuPdPt alloy nanostructures fabricated with the Ag_0.46_Au_0.18_Pd_0.18_Pt_0.18_ quad-layers between 3.4 and 85 nm. Based on the various size and surface configuration of MNPs, distinctive LSPR responses were demonstrated in terms of extinction, reflectance and transmittance spectra. The reflectance and transmittance spectra were directly measured from the samples while the extinction was extracted followed by the relation: extinction (%) = 100%—(reflectance + transmittance) %. In addition, the local E-field and extinction of alloy NPs on sapphire were simulated using the FDTD solutions. The dielectric constants for specific alloy compositions were averaged from the dielectric constant of pure Au, Pd and Pt based on the at. % fraction. For example, based on the ellipsometry measurement for a binary material [20,26], i.e. Ag-Pt, the dielectric constant varies along with the averaged value of at. % fraction. Thus, the Au-Pt dielectric constant was constructed by averaging based on the at. % fraction. Then, the dielectric constant for AuPtPd was again averaged based on the at. % fraction. Generally, the extinction spectra of AuAgPd alloy NPs were gradually increased along with the increased thickness of quad-layers as in Fig 3(a), which can be due to the increased absorption by the larger NPs. Although the extinction spectra generally exhibited two peaks in Fig 3(a), i.e. one in the UV and another in the VIS region, the excitation of LSPR mode and wavelength can vary depending on the size and configurations of NPs. The LSPR bands in the UV and VIS can be induced by the quadrupolar (QP) and dipolar (DP) resonances respectively with the smaller size of AuPdPt NPs [33] and they can be caused by the multipolar (MR) and higher-order (HO) resonance modes with the larger size [34]. Thus, depending upon the size of tri-metallic NPs, the LSPR analysis can be divided into two regimes: i.e. with the smaller NPs (< 250 nm in diameter) between 3.4 and 17 nm deposition thickness and with the larger NPs (> 250 nm) between 25.5 and 85 nm. The LSPR peaks of AuAgPd alloy NPs were significantly altered based on the size and uniformity variation. As shown in Fig 3(a-1), the small alloy NPs fabricated between 3.5 and 17 nm quad-layers demonstrated two distinct QP and DP resonance peaks. The QP and DP peaks were gradually enhanced and red-shifted with the formation of relatively larger and much uniform alloy NPs fabricated with 8.5 nm quad-layer. In addition, the contour map of extinction spectra in Fig 3(d) clearly shows the intensity increment and red-shift. However, as the average size of alloy NPs was increased ~ 300 nm and density was decreased with the thicker (17 nm) quad-layers films, the LSPR peak aspect ratio was found to be slightly reduced with the red shift in position. The red shift of LSPR peak can be due to the size increment while the aspect ratio may vary depending upon the forward or backward scattering behavior of NPs. In specific, the smaller NPs have strong scattering in the backward direction whereas that of large one scatters in the forward direction [35]. When the intermediate size of NPs are formed such as with the 17 nm quad-layer, the scattering in both directions may be very high, which can increase reflectance and transmittance at resonance wavelength, giving a low extinction as seen in Fig 3(a)–3(c). In the case of larger NPs fabricated with the film thickness between 25.5 and 85 nm, the HO and MP resonance peaks were gradually enhanced and red shifted with the increased size of alloy nanostructures, which can be clearly observed in contour map in Fig 3(e). Furthermore, the extinction peak width at 460 nm in Fig 3(a-3) and 3(f) clearly demonstrate the peak narrowing with the smaller and uniform NPs up to 8.5 nm thickness whereas wider with the larger NPs [35]. The 25.5 nm sample is on top likely due to the intermediate scattering behavior that gives a wide spectrum distribution or low aspect ratio as mentioned. The e-field distributions for the typical semi-spherical and elongated AuPdPt alloy NPs are presented in Fig 3(g)–3(j). In addition, the simulated extinction spectra along with the e-field vector are shown in S4 Fig, which showed the broad extinction shoulder in the UV-VIS region. For both configurations of tri-metallic NPs, the e-field was also found to be stronger in the VIS region due to the MP resonance as displayed in Fig 3(h) and 3(j). It was also observed that the e-field was mostly confined at the boundaries and sharp edges of the NPs. The corresponding reflectance spectra are presented in Fig 3(b), which also demonstrated two reflectance dips at UV and VIS depending upon the size and various resonance modes as discussed. The small NPs generally exhibited flatter shape or minor dips, which can be correlated to the strong backward scattering. Whereas, the extinction dips were much enhanced and prominent with the larger alloy NPs. This indicates the increased absorption due to the LSPR of larger alloy NPs. In addition, the transmittance spectra were also studied as presented in Fig 3(c). In the case of smaller alloy NPs, the transmittance exhibited dips in the UV and VIS region corresponding to the QP and DP as discussed. However, larger alloy NPs generally showed a flat response, which can be likely due to the strong forward scattering [36].

**Fig 4:**
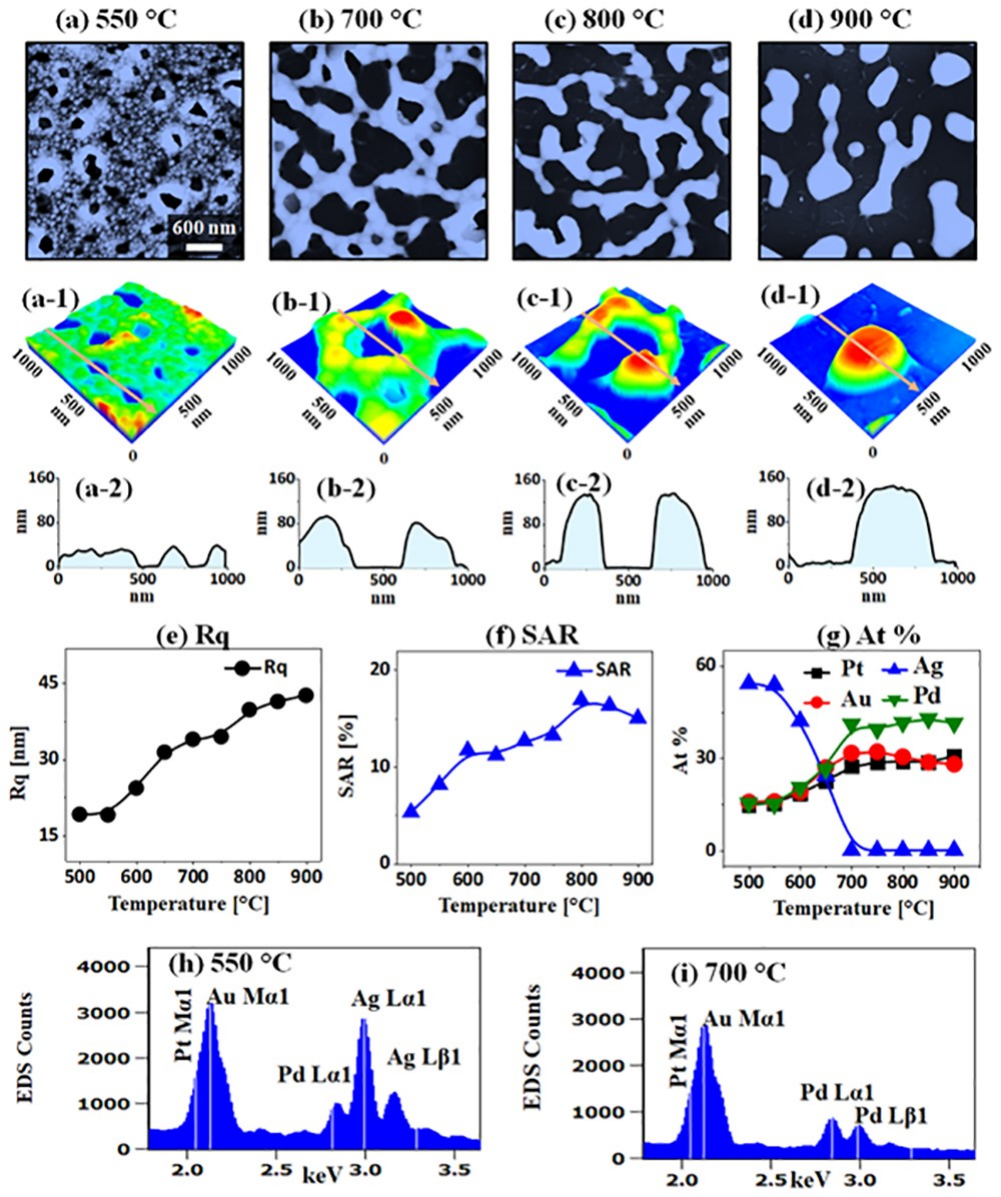
Dewetting of the AgAuPdPt and AuPdPt alloy nanostructures with the Ag_24 nm_ / Au_9 nm_ / Pd_9 nm_ / Pt_9 nm_ quad-layer films by annealing between 550 and 900 °C for 120 s. (a)–(d) AFM top-views of 3 × 3 μm^2^. (a-1)–(d-1) Magnified AFM side-views of 1 × 1 μm^2^. (a-2)–(d-2) Cross-sectional line-profiles. (e)–(f) Plots of Rq and SAR corresponding alloy nanostructures as a function of annealing temperature. (g) Summary plot of at. % of Ag, Au, Pd and Pt. (h)–(i) EDS spectra of the samples annealed at 550 and 700 °C.

**Fig 5:**
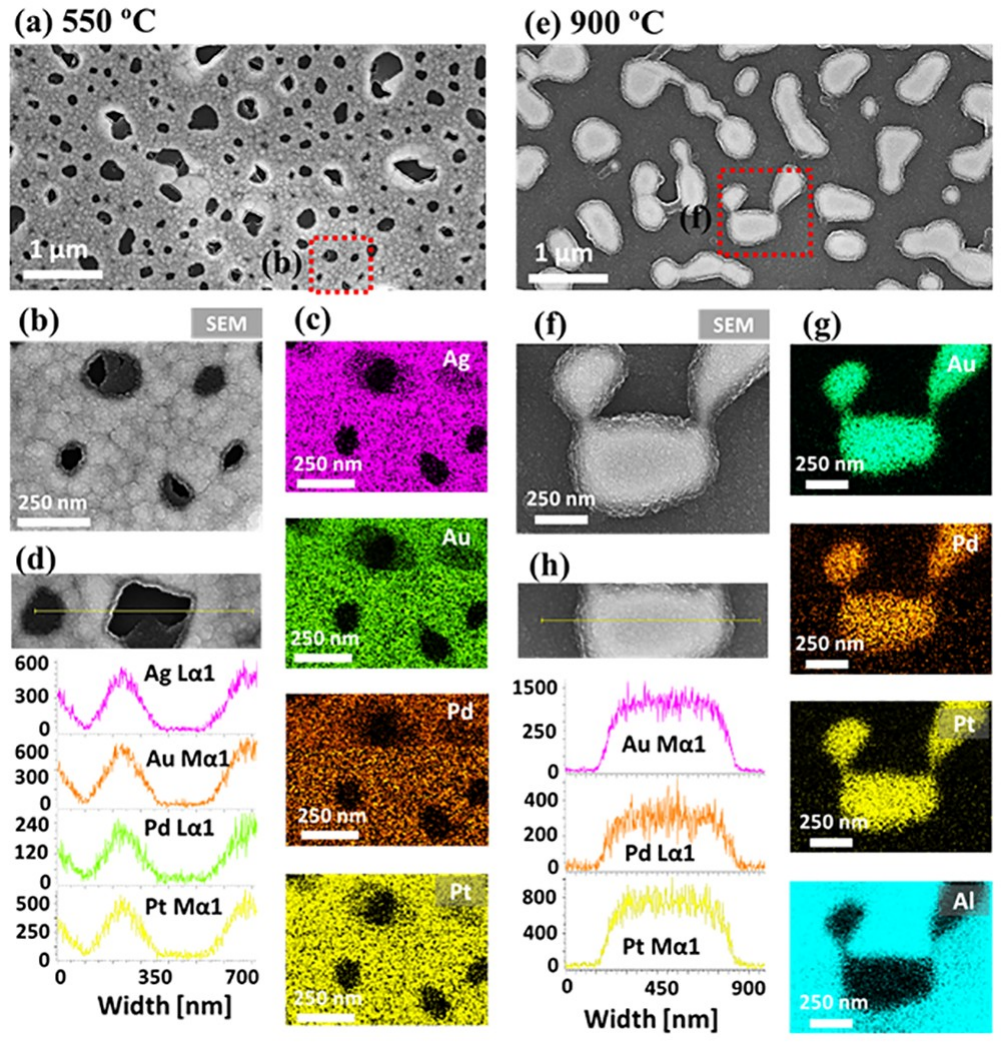
Elemental analysis of the AgAuPdPt and AuPdPt alloy nanostructures with the Ag_24 nm_ / Au_9 nm_ / Pd_9 nm_ / Pt_9 nm_ quad-layer films by annealing between 550 and 900 °C. (a) SEM image of AgAuPdPt nanoclusters at 550 °C. (b)–(c) Enlarged SEM image and the elemental phase maps of Ag, Au, Pd and Pt. (d) EDS line profiles across the typical region. (e) SEM image of the AuPdPt alloy NPs at 900 °C. (f)–(g) Enlarge SEM image and the elemental phase maps of Au, Pd, Pt and Al. (h) EDS line profile across the AuPdPt alloy NP.

Fig 4 shows the effect of annealing temperature (Ta) on the evolution of alloy NPs with the Ag_24 nm_ / Au_9 nm_ / Pd_9 nm_ / Pt_9 nm_ (51 nm in total thickness) quad-layers by annealing between 500 and 900 °C for 120 s. The large-scale AFM and SEM images are presented in S5 and S6 Figs. Generally, the dewetting of quad-layers showed the void nucleation, coalescence, network-like nanoclusters, and isolated NPs as clearly seen in AFM tops-views. The atomic intermixing at different metal interfaces of a multi-layer film can make fully intermixed or alloy layered at a lower temperature. Consequently, the various stages of dewetting such as voids nucleation, void growth, nanoclusters agglomeration, and nanocluster fragmentation can be attained as the Ta increases gradually [37]. In this set, generally, the large size alloy nanoclusters and NPs were obtained with a wide range of coverage. As shown in Fig 4(a), the large voids of ~ 300 nm width and 40 nm depth were observed on the quad-layer at 550 °C. At lower Ta, there were very small voids and granular structures in S5 and S6 Figs, which clearly suggests the initial stage of the dewetting process. The process can be attributed to the coalescence of nearby small voids in order to minimize the interface energy of the system [38]. Meanwhile, the void rims started growing larger due to the accelerated diffusion of atoms around the dewetting fronts. Along with the increased Ta up to 700 °C, the voids were expanded ~ 500 nm and network-like alloy nanoclusters resulted. The Rq and SAR were also consistently increased with temperature as shown in Fig 4(e) and 4(f). At the same time, the growth of alloy nanostructures can be largely affected by the Ag atom sublimation, which was confirmed by the elemental analysis as displayed in Fig 4(g)–4(i), S7 Fig and Fig 5. In specific, all four elements (Ag, Au, Pd, Pt) were presented in the samples up to 550 °C as shown by the summary plot of at. % and EDS spectra in Fig 4(g) and 4(h). However, it was also observed that at. % of Ag was sharply decreased between 550 and 650 °C, which clearly implies the desorption of Ag atoms due to the sublimation. In addition, in order to confirm the alloy formation of deposited elements, the sample annealed at 550 °C was investigated with the elemental mapping as shown in Fig 5(a)–5(d). The SEM image of the partially dewetted nanocluster layer and elemental mapping of Ag, Au, Pd and Pt matched well as displayed in Fig 5(b)–5(c). Furthermore, the EDS line profile through the nanocluster region clearly depicts the homogeneous distribution of elements. At the increased temperature at 700 °C, the at % of Ag became ~ 0% as clearly seen in Fig 4(g) and 4(i), suggesting the complete sublimation of Ag and formation of tri-metallic AuPdPt nanostructures. Consequently, the network-like AuPdPt nanoclusters showed fluctuation in width at a certain area and started to fragment at 800 °C as displayed in Fig 4(c)–4(c-1). This process can be correlated to the variation in the surface energy and Rayleigh-like instability as discussed [39,40]. Finally, at 900 °C, the isolated NPs were transformed into the large elongated configurations as observed in Fig 4(d)–4(d-1). The typical height and diameter of these NPs were about 600 and 160 nm respectively and the Rq and SAR were also gradually increased for the nanoclusters. However, the SAR was found to be slightly decreased above 850 °C likely due to the reduced density of NPs. In terms of elemental composition, it remained consistent between 700 and 900 °C as there was no sublimation loss of Au, Pd and Pt atoms while Ag atoms were already sublimated. The deatiled elemental distribution of AuPdPt alloy NPs at 900 °C is presented in Fig 5(e)–5(h), which clearly show the homogeneously distributed Au, Pd and Pt atoms in the NPs and absence of Ag atoms. The evolution of self-assembled alloy nanostructures fabricated with the Ag_12 nm_ / Au_4.5 nm_ / Pd_4.5 nm_ / Pt_4.5 nm_ (25.5 nm in total thickness) quad-layers showed a similar trend to the the Ag_24 nm_ / Au_9 nm_ / Pd_9 nm_ / Pt_9 nm_ (51 nm in total thickness) quad-layers in S12–S16 Figs along with the morphology, elemental optical analysis.

**Fig 6:**
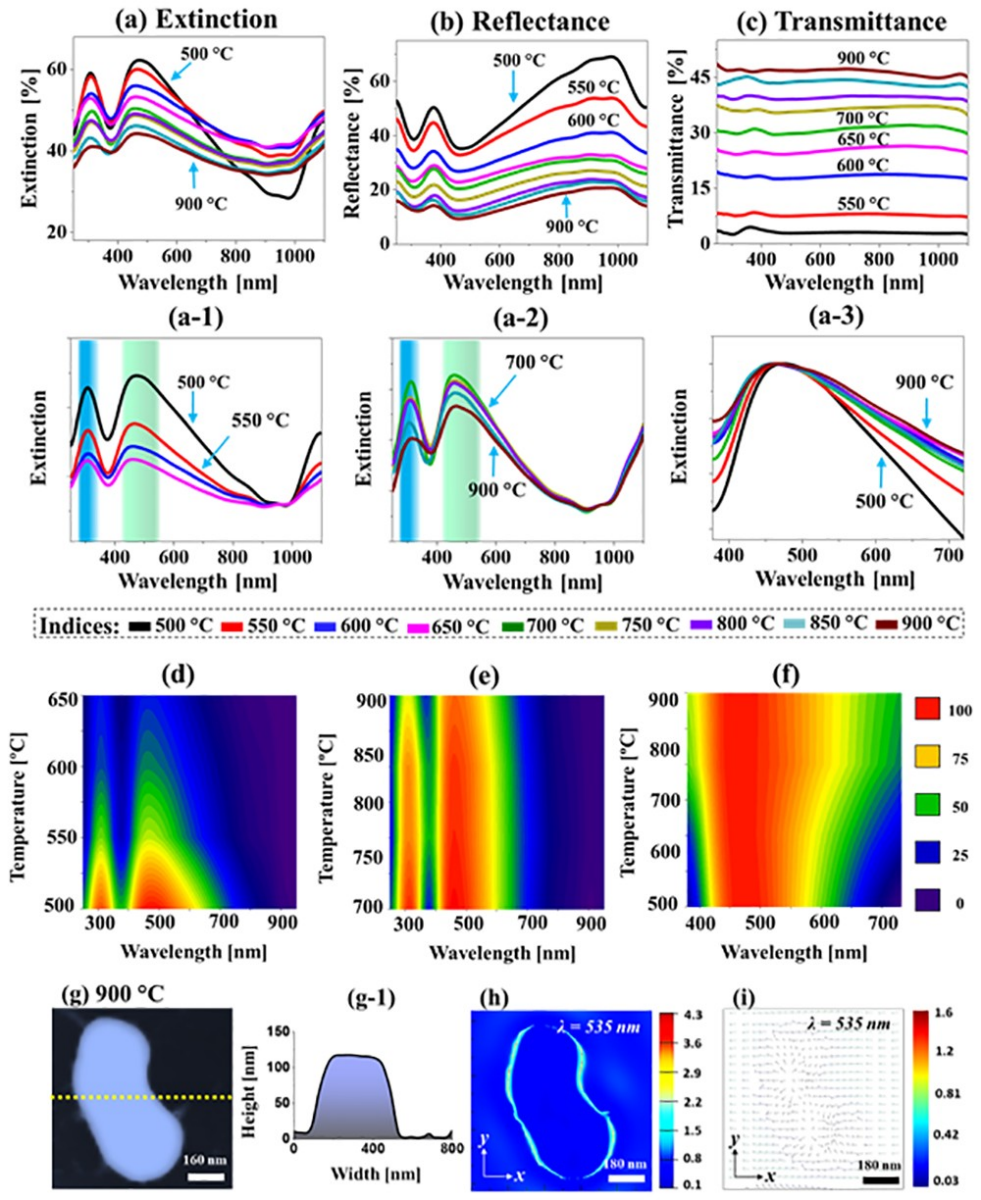
Optical properties of large AgAuPdPt and AuPdPt alloy nanostructures fabricated with the Ag_24 nm_ / Au_9 nm_ / Pd_9 nm_ / Pt_9 nm_ quad-layers. (a)–(c) Extinction, reflectance and transmittance spectra. (a-1)–(a-3) Normalized extinction spectra. (d)–(f) Contour maps of the extinction spectra in (a-1)–(a-3). (g) AFM image of the typical AuPdPt NP. (g-1) Cross-sectional line profile of the typical NP. (h)–(i) E-field distribution at resonance wavelengths in xy-plane.

Fig 6 shows the LSPR properties of large AgAuPdPt and AuPdPt nanocluster fabricated with the Ag_24 nm_ / Au_9 nm_ / Pd_9 nm_ / Pt_9 nm_ quad-layers. The extinction spectra in Fig 6(a) generally exhibited two intense LSPR peaks at UV and VIS region for all the samples, which can be correlated to the excitation of HO and MP resonance bands because of the large size and wide size distribution as discussed [34]. The LSPR peaks were gradually attenuated with the increased Ta as shown in Fig 6(a-1) and 6(a-2) likely due to the sublimation of Ag atoms and average size reduction. Meanwhile, the LSPR peaks were gradually blue-shifted between ~ 480 and 450 nm for the samples annealed between 500 and 900 °C due to the average size reduction of alloy NPs. The extinction peak intensity and shift trends are clearly shown by the contour maps in the Fig 6(d)–6(e). It was also observed that the LSPR peak width was widened at increased Ta for the AuPdPt NPs in Fig 6(a-3) and 6(f). The LSPR property of typical AuPdPt alloy NP was further studied by the FDTD simulation as presented in Fig 6(g)–6(i) and S10 Fig. As displayed in S10 Fig, the extinction spectra exhibited a shoulder in the UV-VIS region, which can be correlated to the MP resonance of large and irregular alloy NP. The e-field was mostly confined at the edge of NPs and substrate and had multiple e-field vector directions corresponding to the MP resonance modes. In addition, the reflectance spectra in Fig 6(b) demonstrated two dips, corresponding to the HO and MP resonance modes. The reflectance dip intensity was gradually attenuated at increased Ta due to the size reduction of alloy NPs along with the Ag sublimation. Furthermore, the transmittance in Fig 6(c) generally showed flatter response for all samples likely due to the stronger forward scattering by the large alloy nanostructures. In terms of average reflectance and transmittance, similarly, they showed the opposite trend depending on the surface coverage of alloy nanostructures.

**Fig 7:**
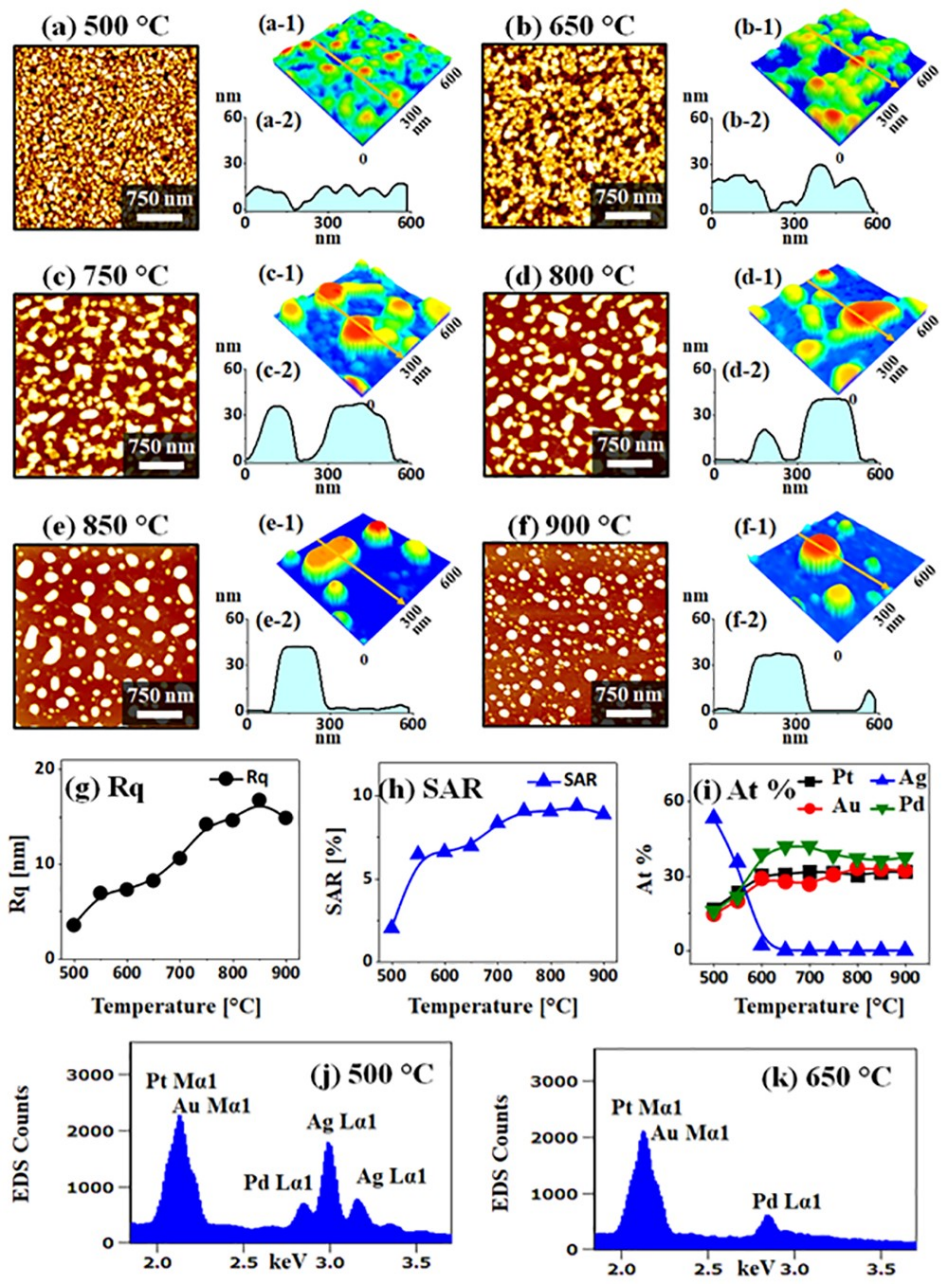
Evolution of small alloy NPs on sapphire by the annealing of Ag_8 nm_ / Au_3 nm_ / Pd_3 nm_ / Pt_3 nm_ at various temperature between 500 and 900 °C for 120 s. (a)–(f) AFM top-views (3 × 3 μm^2^). (a-1)–(f-1) Magnified AFM side-views of 1 × 1 μm^2^. (g)–(h) Summary plots of Rq and SAR. (i) Plot of elemental composition in terms of atomic (at) % of Ag, Au, Pd and Pt. (j)–(k) EDS spectra of the samples annealed at 500 and 650 °C.

Fig 7 shows the evolution of small size AgAuPdPt and AuPdPt NPs with the Ag_8 nm_ / Au_3 nm_ / Pd_3 nm_ / Pt_3 nm_ quad-layers under an identical growth condition as in the previous set. In general, the transformation of irregular-connected to widely spaced alloy NPs was observed with increasing Ta with the fixed quad-layer thickness. It was also observed that the size of the NPs was significantly decreased the density increased at the specific Ta as compared to the previous set. As shown in Fig 7(a)–7(b), the void nucleation and growth was observed at 500 and 650 °C. Along with the increased Ta, the voids and nanoclusters were grown larger due to the enhanced atomic diffusion and agglomeration. Since the layered nanostructures were gradually agglomerated into the compact nanoclusters, the Rq and SAR were also correspondingly increased between 500 and 650 °C as shown in Fig 7(g) and 7(h). Similar to the previous set, the growth of alloy NPs was affected by the Ag atom sublimation as shown in Fig 7(i)–7(k). From the summary plots of at %, the Ag at % was decreased from ~ 58 to 0% when the Ta was varied between 500 and 650 °C, indicating the sharp sublimation of Ag atoms in the temperature regime. At the higher Ta, i.e above 650 °C, the NPs only consisted of Au, Pd and Pt atoms as clearly depicted by the at % plot and EDS spectra. The surface morphology of AuPdPt alloy NPs was gradually evolved further by the transformation from the irregular-connected to the isolated semi-spherical as observed in Figs 7(c)–4(f). The average size of isolated alloy NPs was increased up to ~ 40 and 260 nm in terms of height and diameter respectively as shown in the corresponding 3-D side-views and line profiles. Since the Ag was already sublimated, the evolution in this temperature range can be correlated to the diffusion and agglomeration of Au, Pd and Pt atoms in the alloy form. At the same time, the large nanoclusters can be fragmented and became regular in shape based on the Rayleigh-like instability and surface energy minimization [33, 34]. Since the elongated alloy NPs were fragmented, the areal density of NPs was gradually increased between 750 and 900 °C. The Rq and SAR of isolated alloy NPs at higher Ta also showed the consistent increment as displayed in Fig 7(g)–7(h). This again indicates the gradual increment of the average height as well as the surface area of the nanostructures. The elemental composition of various alloy nanostructures between 650 and 900 °C was found to be almost similar, which indicates the same elemental composition of the AuPdPt alloy nanostructures.

**Fig 8:**
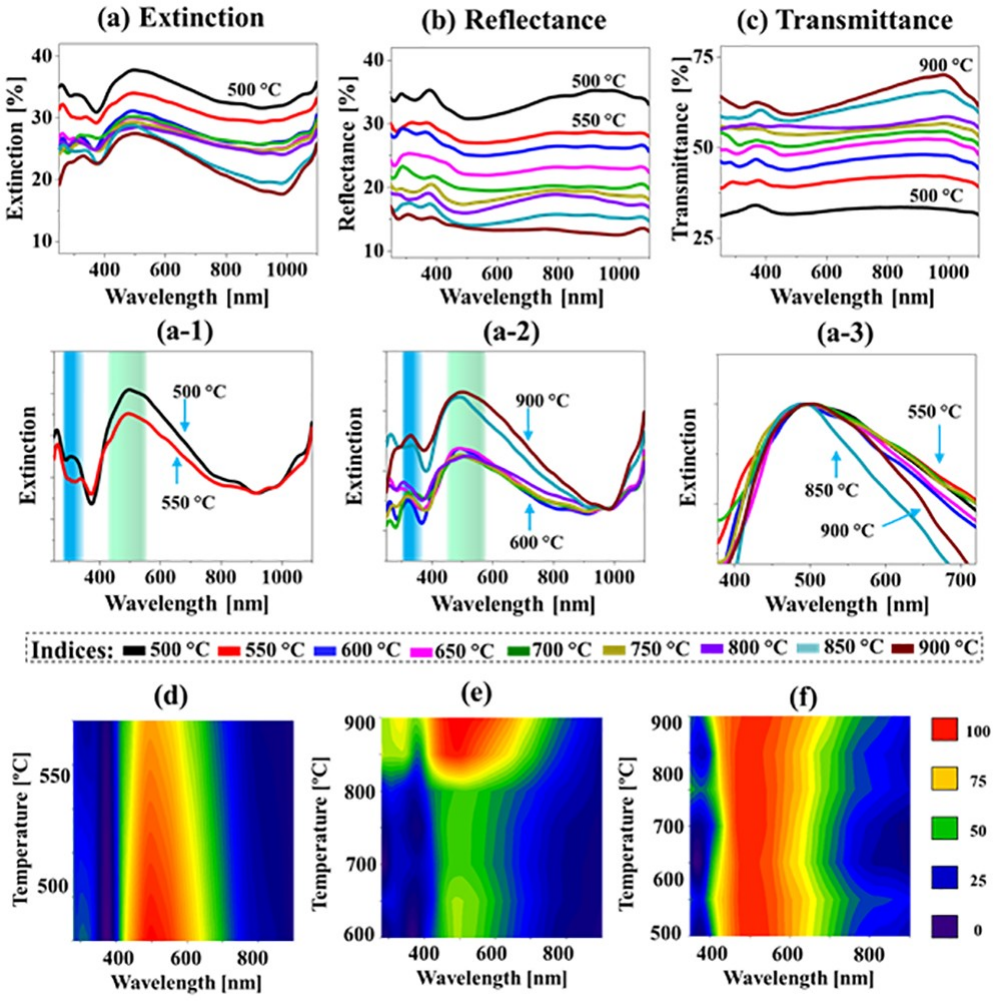
Optical properties of small alloy NPs fabricated with the Ag_8 nm_ / Au_3 nm_ / Pd_3 nm_ / Pt_3 nm_ quad-layers. (a)–(c) Extinction, reflectance and transmittance spectra. (a-1)–(a-3) Normalized extintion spectra. (d)–(f) Contour maps of the extinction spectra in (a-1)–(a-3).

Fig 8 shows the LSPR properties of the quad-metallic AgAuPdPt and tri-metallic AuPdPt alloy NPs fabricated with the Ag_8 nm_ / Au_3 nm_ / Pd_3 nm_ / Pt_3 nm_ quad-layer films at different Ta. Generally, the extinction spectra in Fig 8(a) demonstrated two distinct LSPR bands in the UV and VIS region, which can be induced by the HO and MP resonance mode respectively as discussed [33]. The LSPR of alloy nanostructures were correspondingly altered based on the composition and morphology at increased Ta. For instance, the quad-metallic AgAuPdPt nanoclusters below 600 °C exhibited the stronger LSPR bands while the tri-metallic AuPdPt nanostructure above 650 °C showed the comparatively weaker peaks. Although the overall size of alloy NPs was slightly increased between 500 and 600 °C, the extinction peaks were attenuated as shown in Fig 8(a-1) likely due to the gradual Ag sublimation. The corresponding contour map in Fig 8(d) clearly show the intensity loss at high temperature. However, with the formation of semispherical AuPdPt NPs at higher Ta, the LSPR peak intensity was found to be enhanced as displayed in Fig 8(a-2) and 8(e). This can be correlated to the improvement on size and uniformity of the isolated alloy NPs. Meanwhile, the LSPR can be mainly contributed by the QP and DP resonance modes in the UV and VIS regions with the compact semispherical configuration and improved uniformity of tri-metallic AuPdPt NPs. Thus, the VIS absorption peak can be narrowed between 600 and 800 °C due to the improved uniformity of alloy NPs at higher Ta [37] as shown in Fig 8(a-3) and 8(f). However, as the segmentation of large NPs yielded the wide distribution of size above 850 °C, the extinction peaks were again broadened. Furthermore, the corresponding reflectance spectra of multimetallic alloy NPs are presented in Fig 8(b), which commonly exhibited the reflectance dips in the UV and VIS region corresponding to the HO and MP resonance modes [38]. The transmittance spectra analysis of the corresponding samples is presented in Fig 8(c), which also clearly demonstrated two dips at UV and VIS regions due to the HO and MP resonance modes as discussed. The dip in the VIS region was gradually enhanced and narrowed with the increased Ta due to the size and uniformity improvement of NPs. Furthermore, the average transmittance was gradually increased as a function of annealing temperature based on the reduced surface coverage.

## Conclusions

In summary, various multi-metallic (Au, Ag, Pd and Pt) alloy NPs were successfully demonstrated on sapphire (0001) by utilizing a solid-state dewetting of Ag/Au/Pd/Pt quad-layers. By the systematic control of annealing temperature and film thickness, the size, shape, density and elemental composition of alloy NPs were readily tuned. The Ag sublimation turned to be significantly affecting the growth multi-metallic NPs in terms of NP configuration and elemental composition as well as the LSPR properties. Specifically, the control of initial film thickness between 3.4 to 85 nm at 850 °C resulted in the formation of various configurations of tri-metallic AuPdPt NPs: i.e. from the tiny and dense to the large and widely spaced NPs. By the systematic control of annealing temperature, the quad-metallic AgAuPdPt and tri-metallic AuPdPt NPs of various size and density were fabricated. The LSPR properties of various alloy NPs showed strong LSPR response in the UV and VIS regions. Depending upon the size and elemental compositions, the LSPR band were varied in terms of intensity and peak positions. Specific NPs were simulated for the e-field distributions at resonance wavelengths.

## Supporting information

S1 Fig Surface morphology and optical properties of degassed bare sapphire (0001): (a)–(a-2) AFM top view, side-view and corresponding line-profile. (b) Reflectance spectrum. (c) Transmittance spectrum.
(DOCX)

S2 Fig Surface morphology of as-deposited Ag_0.46_Au_0.18_Pd_0.18_Pt_0.18_ quad-layer samples with a thickness between 3.4 and 85 nm. (a)–(e) are AFM top-views 3 × 3 μm^2^. (a-1)–(e-1) Magnified AFM side-views of 1 × 1 μm^2^. (a-2)–(e-2) Cross-sectional line-profiles. (f) Plots of Rq and SAR corresponding samples, showing a gradual increase along with increased deposition thickness due to the increased surface corrugation. In this experiment, all the metallic films were deposited with an identical growth rate of 0.05 nm/s at the ionization current 3 mA, i.e. 20 s = 1 nm. In the case of Ag_8 nm_ / Au_3 nm_ / Pd_3 nm_ / Pt_3 nm_ quad-layer films (total thickness (t) = 17 nm), initially 8 nm of Ag film was deposited on sapphire (0001) and then 3 nm of Au, 3 nm of Pd and 3 nm of Pt films were deposited subsequently atop.
(DOCX)

S3 Fig (a)–(f) EDS spectra of various AuPdPt alloy nanostructures fabricated with the Ag_0.46_Au_0.18_Pd_0.18_Pt_0.18_ quad-layers and total thickness between 3.4 and 85 nm at 850 °C for 120 s. (DOCX)

S4 Fig Finite difference time domain (FDTD) simulation of the typical AuPdPt alloy NP fabricated with the Ag_8 nm_ / Au_3 nm_ / Pd_3 nm_ / Pt_3 nm_ at 750 °C for 120 s. (a) and (e) AFM images of the semispherical and elongated AuPdPt NPs. (b) and (f) simulated extinction spectra of the AuPdPt NPs. (c) and (g) e-field profiles in xy-plane at resonance wavelengths. (d) and (h) e-field vector plots. For the simulation, the typical AFM images were imported in the structure space using surface import. The TFSF source was engaged along the z-direction and the absorption and scattering power were monitored. The extinction was calculated by summing up the absorption and scattering power. The PML boundary condition was adapted in all direction, in which the minimum distance between PML boundary and the structure is greater than 500 nm.
(DOCX)

S5 Fig Various alloy nanostructures fabricated with the Ag_24 nm_ / Au_9 nm_ / Pd_9 nm_ / Pt_9 nm_ quad-layer films at various annealing temperature between 500 and 900 °C for 120 s. (a)–(i) AFM side-views of 3 × 3 μm^2^. (a-1)–(i-1) Corresponding cross-sectional line-profiles.
(DOCX)

S6 Fig SEM images of alloy nanostructures fabricated with the Ag_24 nm_ / Au_9 nm_ / Pd_9 nm_ / Pt_9 nm_ quad-layer films at various annealing temperature between 500 and 900 °C for 120 s. (DOCX)

S7 Fig EDS spectral analysis of various alloy nanostructure fabricated with the Ag_24 nm_ / Au_9 nm_ / Pd_9 nm_ / Pt_9 nm_ quad-layer films at various annealing temperatures as labeled. (DOCX)

S8 Fig Elemental analysis of the AgAuPdPt with the Ag_24 nm_ / Au_9 nm_ / Pd_9 nm_ / Pt_9 nm_ quad-layer films by annealing between 550 °C. (a) SEM image. (b) Enlarged SEM image. (c) EDS line profile across the nanocluster and void regions. (d)–(h) Elemental phase mapping of Ag, Au, Pd, Pt and Al.
(DOCX)

S9 Fig Elemental analysis of the AuPdPt with the Ag_24 nm_ / Au_9 nm_ / Pd_9 nm_ / Pt_9 nm_ quad-layer films by annealing between 900 °C. (a) SEM image. (b) Enlarged SEM image. (c) EDS line profile across the AuPdPt alloy NP. (d)–(h) Elemental phase mapping of Au, Pd, Pt, Al and O.
(DOCX)

S10 Fig Finite difference time domain (FDTD) simulation of the typical AuPdPt alloy NP fabricated with the Ag_24 nm_ / Au_9 nm_ / Pd_9 nm_ / Pt_9 nm_ at 750 °C for 120 s. (a) AFM image. (b) Simulated extinction plot of the corresponding AuPdPt NP. (c) E-field profile in xy-plane at resonance wavelength. (d) E-field vector plots.
(DOCX)

S11 Fig (a)–(f) EDS spectra of AgAuPdPt and AuPdPt alloy nanostructures fabricated with the Ag_8 nm_ / Au_3 nm_ / Pd_3 nm_ / Pt_3 nm_ between 500 to 900 °C for 120 s. (DOCX)

S12 Fig Evolution of AuPdPt alloy nanostructures from connected to isolated NPs on saaphire (0001) by the systemetic control of annealing temperature between 500 and 900 °C for 120 s with Ag_12 nm_ / Au_4.5 nm_ / Pd_4.5 nm_ / Pt_4.5 nm_ films. (a)–(i) AFM side-views of 5 × 5 μm^2^. (a-1)–(i-1) Corresponding cross-sectional line-profiles.
(DOCX)

S13 Fig Evolution of self-assembled alloy nanostructures fabricated with the Ag_12 nm_ / Au_4.5 nm_ / Pd_4.5 nm_ / Pt_4.5 nm_ quad-layers. (a)–(d) SEM images of corresponding alloy nanostructures between 600 and 900 °C. (a-1)–(d-1) AFM top-views of 3 × 3 μm^2^. (a-2)–(d-2) Magnified AFM side-views of 1 × 1 μm^2^. (a-3)–(d-3) Cross-sectional line-profiles. (e)–(g) Plots of average roughness (Ra), RMS roughness (Rq) and surface area ratio (SAR) of corresponding alloy nanostructures as a function of annealing temperature.
(DOCX)

S14 Fig SEM images of alloy nanostructures fabricated with the Ag_12 nm_ / Au_4.5 nm_ / Pd_4.5 nm_ / Pt_4.5 nm_ quad-layers at various annealing temperatures as labeled. (DOCX)

S15 Fig EDS spectra of various alloy nanostructure fabricated with the Ag_12 nm_ / Au_4.5 nm_ / Pd_4.5 nm_ / Pt_4.5 nm_ films at various annealing temperatures as labeled. (DOCX)

S16 Fig Optical properties of alloy nanostructures fabricated with the Ag_12 nm_ / Au_4.5 nm_ / Pd_4.5 nm_ / Pt_4.5 nm_ quad-layers at various annealing temperature between 600 and 900 °C. (a) Extinction spectra. (a-1)–(a-2) Normalized extinction spectra. (a-3) Magnified extinction spectra. (b) Reflectance spectra. (b-1)–(b-2) Normalized reflectance spectra. (b-3) Average reflectance. (c) Transmittance spectra. (c-1)–(c-2) Normalized transmittance spectra. (c-3) Average transmittance.
(DOCX)
