# Supplementary material for: Systematic investigation on quad-metallic AgAuPdPt and tri-metallic AuPdPt NPs through the solid-state dewetting of quad-layer Ag/Au/Pd/Pt thin films on c-plane sapphire
Source: PLoS One. 2019 Oct 21;14(10):e0224208. doi: 10.1371/journal.pone.0224208 (PMC6802835; doi:10.1371/journal.pone.0224208)
Supplement: S1 Fig — Surface morphology and optical properties of degassed bare sapphire (0001): (a)–(a-2) AFM top view, side-view and corresponding line-profile. (b) Reflectance spectrum. (c) Transmittance spectrum. (DOCX) [file pone.0224208.s001.docx]

**
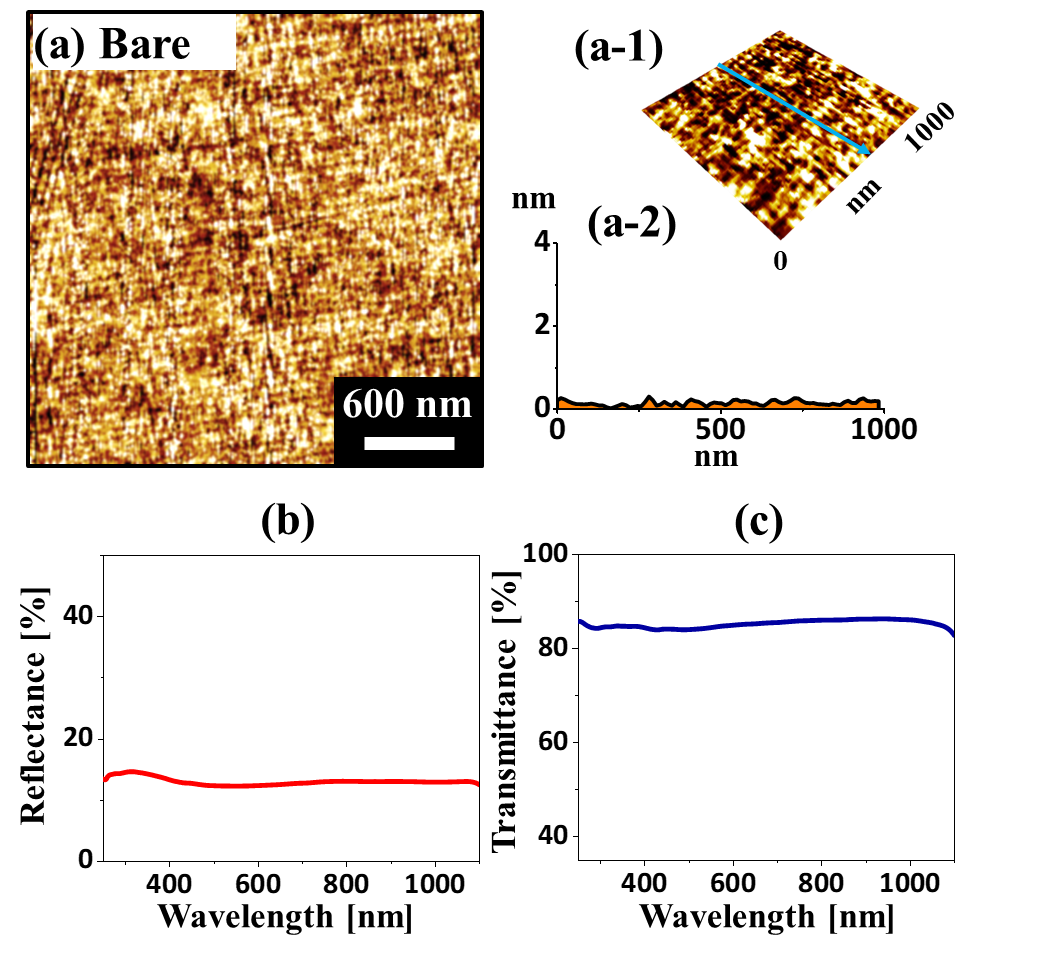
**

**S1 Fig.** Surface morphology and optical properties of degassed bare sapphire (0001) (a) – (a-2) AFM top view, side-view and corresponding line-profile. (b) Reflectance spectrum. (c) Transmittance spectrum.
