# Supplementary material for: Systematic investigation on quad-metallic AgAuPdPt and tri-metallic AuPdPt NPs through the solid-state dewetting of quad-layer Ag/Au/Pd/Pt thin films on c-plane sapphire
Source: PLoS One. 2019 Oct 21;14(10):e0224208. doi: 10.1371/journal.pone.0224208 (PMC6802835; doi:10.1371/journal.pone.0224208)
Supplement: S2 Fig — (a)–(e) are AFM top-views 3 × 3 μm2. (a-1)–(e-1) Magnified AFM side-views of 1 × 1 μm2. (a-2)–(e-2) Cross-sectional line-profiles. (f) Plots of Rq and SAR corresponding samples, showing a gradual increase along with increased deposition thickness due to the increased surface corrugation. In this experiment, all the metallic films were deposited with an identical growth rate of 0.05 nm/s at the ionization current 3 mA, i.e. 20 s = 1 nm. In the case of Ag8 nm / Au3 nm / Pd3 nm / Pt3 nm quad-layer films (total thickness (t) = 17 nm), initially 8 nm of Ag film was deposited on sapphire (0001) and then 3 nm of Au, 3 nm of Pd and 3 nm of Pt films were deposited subsequently atop. (DOCX) [file pone.0224208.s002.docx]

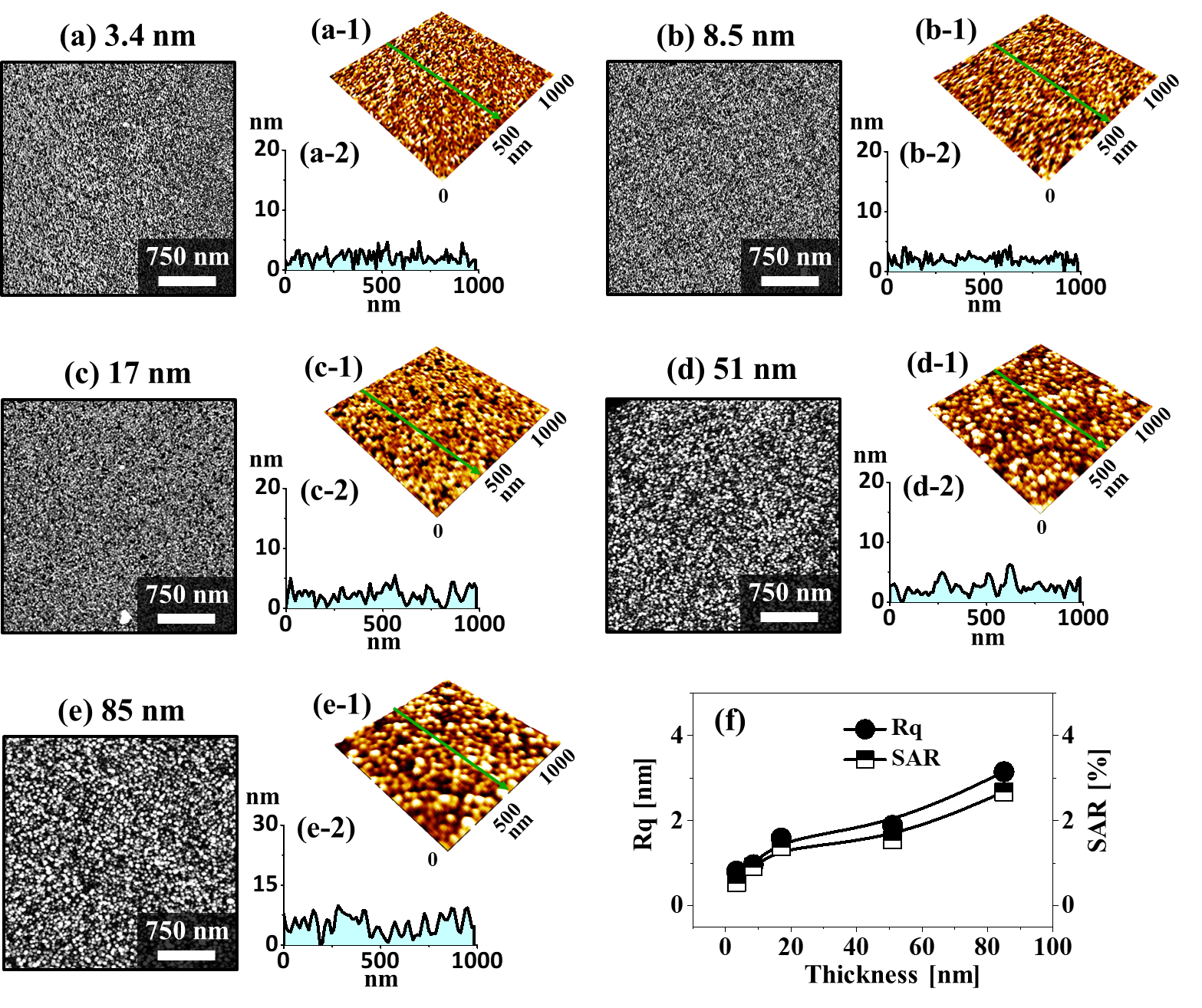


**S2** **Fig.** Surface morphology of as-deposited Ag_0.46_Au_0.18_Pd_0.18_Pt_0.18_ quad-layer samples with a thickness between 3.4 and 85 nm. (a) – (e) are AFM top-views 3 × 3 µm^2^. (a-1) – (e-1) Magnified AFM side-views of 1 × 1 µm^2^. (a-2) – (e-2) Cross-sectional line-profiles. (f) Plots of Rq and SAR corresponding samples, showing a gradual increase along with increased deposition thickness due to the increased surface corrugation. In this experiment, all the metallic films were deposited with an identical growth rate of 0.05 nm/s at the ionization current 3 mA, i.e. 20 s = 1 nm. In the case of Ag_8 nm_ / Au_3 nm_ / Pd_3 nm_ / Pt_3 nm_ quad-layer films (total thickness (t) = 17 nm), initially 8 nm of Ag film was deposited on sapphire (0001) and then 3 nm of Au, 3 nm of Pd and 3 nm of Pt films were deposited subsequently atop.
