# Supplementary material for: Systematic investigation on quad-metallic AgAuPdPt and tri-metallic AuPdPt NPs through the solid-state dewetting of quad-layer Ag/Au/Pd/Pt thin films on c-plane sapphire
Source: PLoS One. 2019 Oct 21;14(10):e0224208. doi: 10.1371/journal.pone.0224208 (PMC6802835; doi:10.1371/journal.pone.0224208)
Supplement: S3 Fig — (DOCX) [file pone.0224208.s003.docx]

**
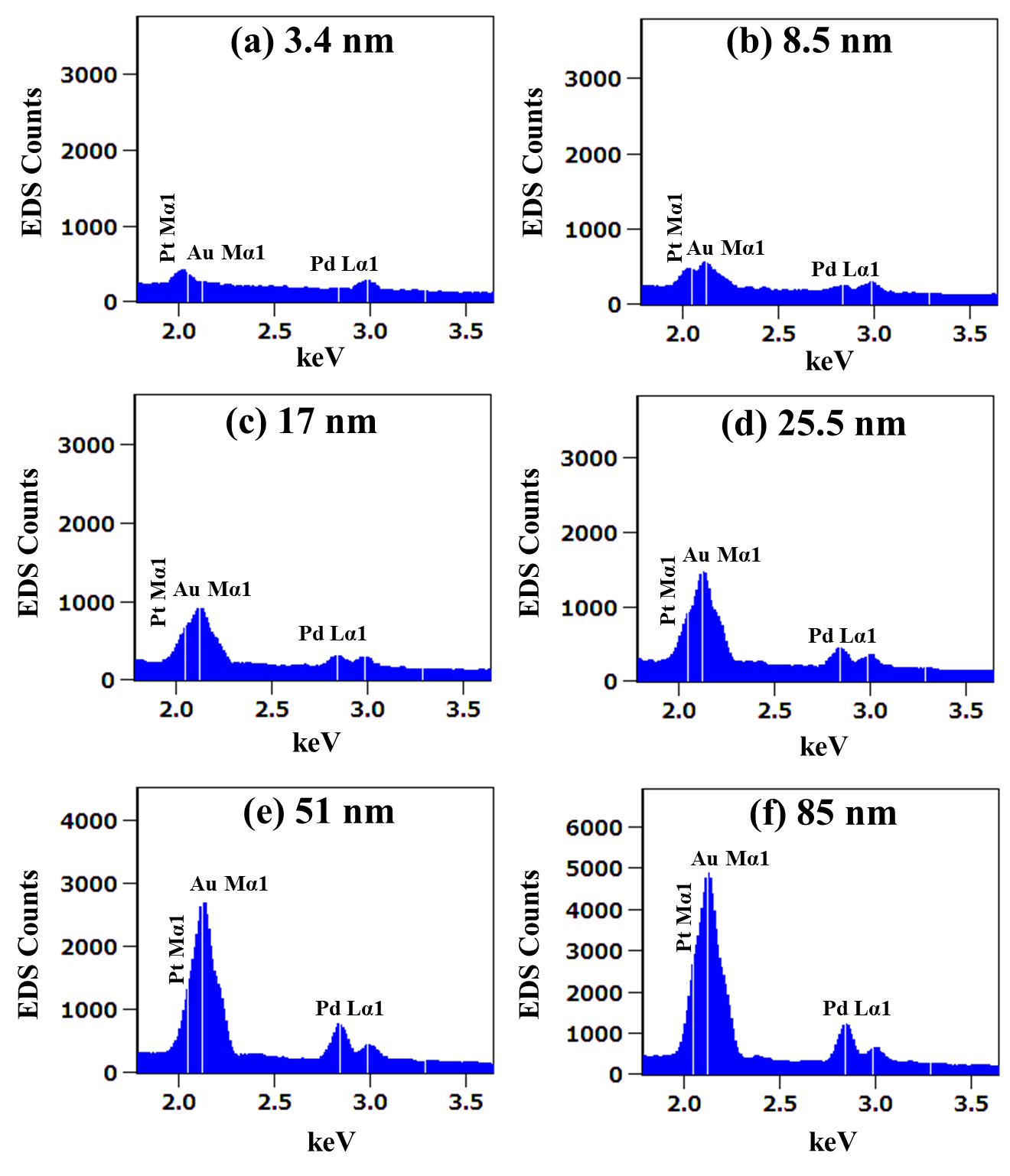
**

**S3 Fig.** (a) – (f) EDS spectra of various AuPdPt alloy nanostructures fabricated with the Ag_0.46_Au_0.18_Pd_0.18_Pt_0.18_ quad-layers and total thickness between 3.4 and 85 nm at 850 °C for 120 s.
