# Supplementary material for: Systematic investigation on quad-metallic AgAuPdPt and tri-metallic AuPdPt NPs through the solid-state dewetting of quad-layer Ag/Au/Pd/Pt thin films on c-plane sapphire
Source: PLoS One. 2019 Oct 21;14(10):e0224208. doi: 10.1371/journal.pone.0224208 (PMC6802835; doi:10.1371/journal.pone.0224208)
Supplement: S4 Fig — (a) and (e) AFM images of the semispherical and elongated AuPdPt NPs. (b) and (f) simulated extinction spectra of the AuPdPt NPs. (c) and (g) e-field profiles in xy-plane at resonance wavelengths. (d) and (h) e-field vector plots. For the simulation, the typical AFM images were imported in the structure space using surface import. The TFSF source was engaged along the z-direction and the absorption and scattering power were monitored. The extinction was calculated by summing up the absorption and scattering power. The PML boundary condition was adapted in all direction, in which the minimum distance between PML boundary and the structure is greater than 500 nm. (DOCX) [file pone.0224208.s004.docx]

**
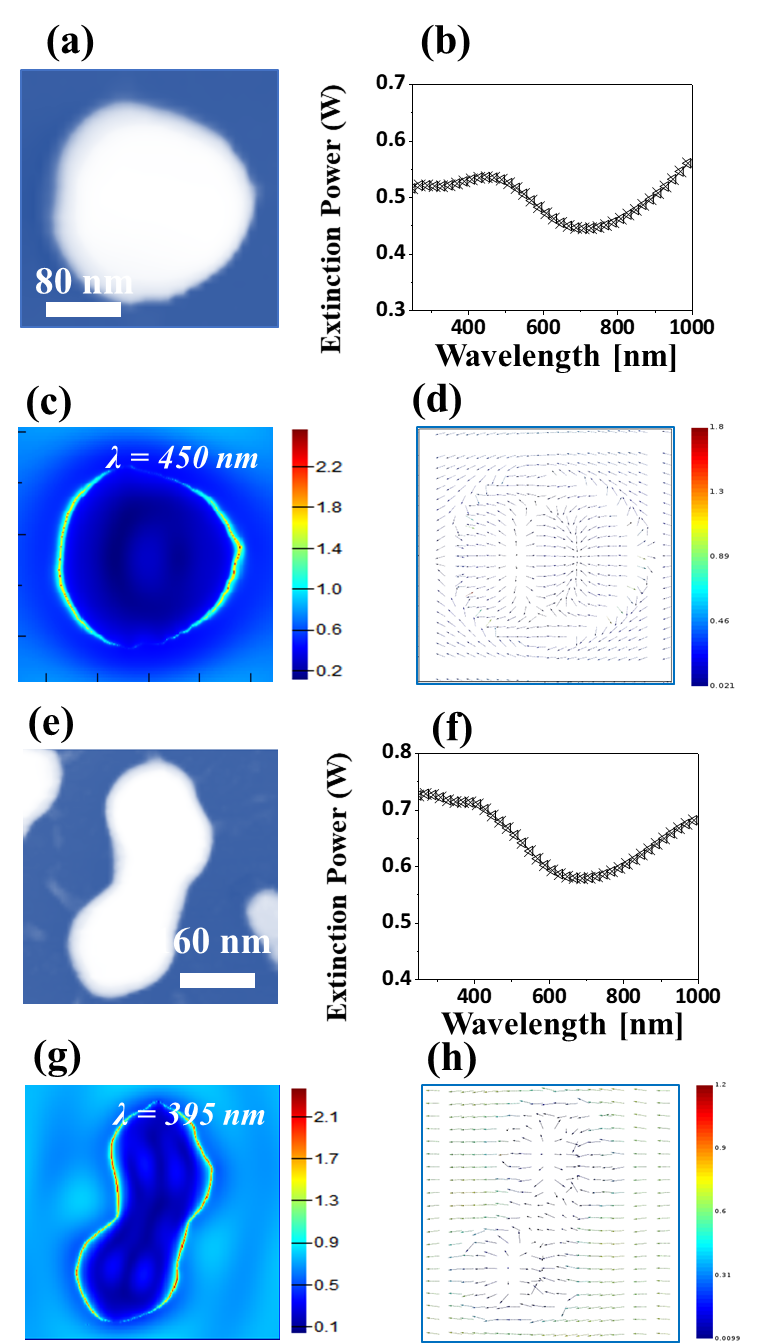
**

**Figure S4:** Finite difference time domain (FDTD) simulation of the typical AuPdPt alloy NP fabricated with the Ag_8 nm_ / Au_3 nm_ / Pd_3 nm_ / Pt_3 nm_ at 750 ^o^C for 120 s. (a) and (e) AFM images of the semispherical and elongated AuPdPt NPs. (b) and (f) simulated extinction spectra of the AuPdPt NPs. (c) and (g) e-field profiles in xy-plane at resonance wavelengths. (d) and (h) e-field vector plots. For the simulation, the typical AFM images were imported in the structure space using surface import. The TFSF source was engaged along the z-direction and the absorption and scattering power were monitored. The extinction was calculated by summing up the absorption and scattering power. The PML boundary condition was adapted in all direction, in which the minimum distance between PML boundary and the structure is greater than 500 nm.
