# Supplementary material for: Systematic investigation on quad-metallic AgAuPdPt and tri-metallic AuPdPt NPs through the solid-state dewetting of quad-layer Ag/Au/Pd/Pt thin films on c-plane sapphire
Source: PLoS One. 2019 Oct 21;14(10):e0224208. doi: 10.1371/journal.pone.0224208 (PMC6802835; doi:10.1371/journal.pone.0224208)
Supplement: S5 Fig — (a)–(i) AFM side-views of 3 × 3 μm2. (a-1)–(i-1) Corresponding cross-sectional line-profiles. (DOCX) [file pone.0224208.s005.docx]

**
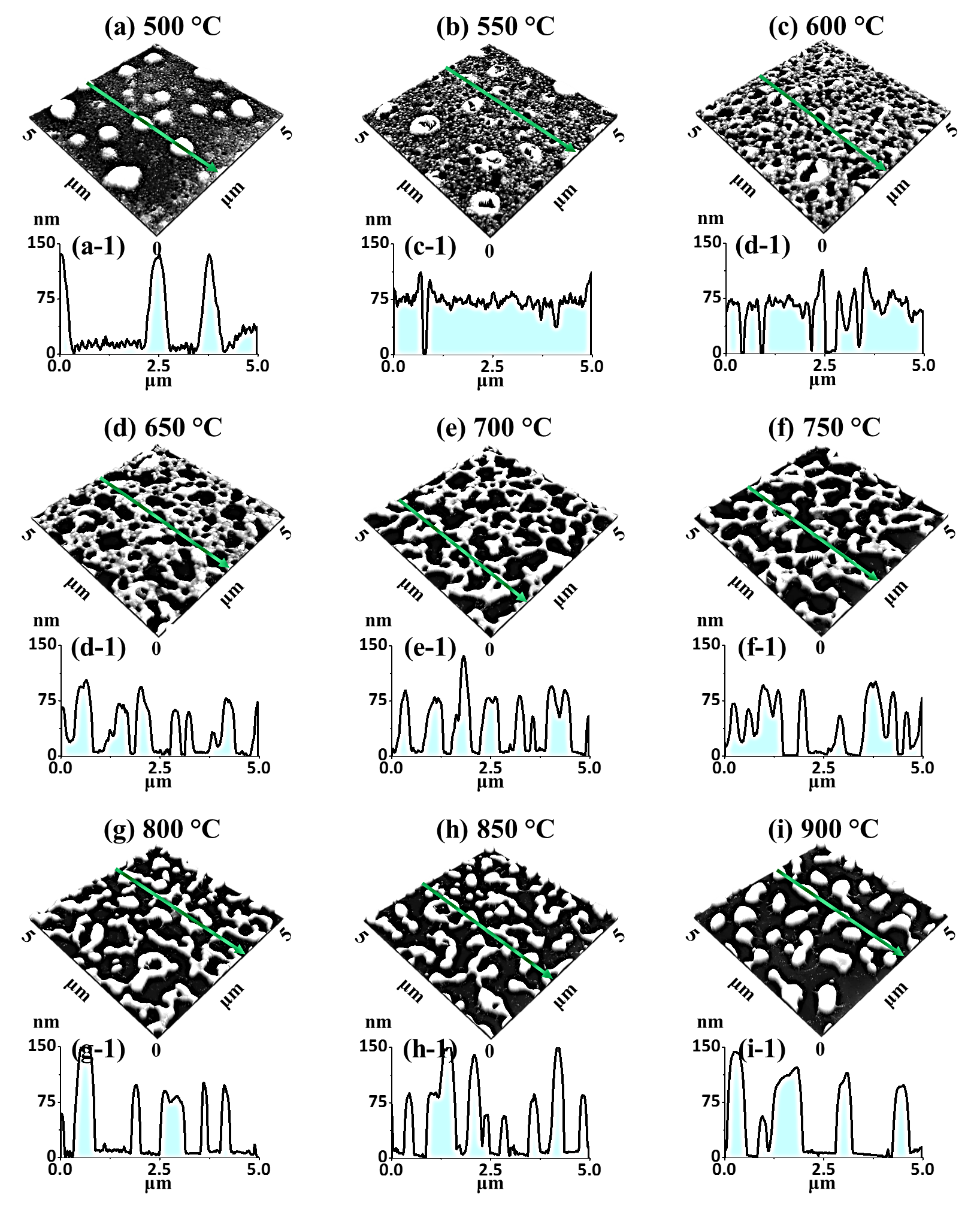
**

**Figure S5:** Various alloy nanostructures fabricated with the Ag_24 nm_ / Au_9 nm_ / Pd_9 nm_ / Pt_9 nm_ quad-layer films at various annealing temperature between 500 and 900 ^o^C for 120 s. (a) – (i) AFM side-views of 3 × 3 µm^2^. (a-1) – (i-1) Corresponding cross-sectional line-profiles.
