# Supplementary material for: Systematic investigation on quad-metallic AgAuPdPt and tri-metallic AuPdPt NPs through the solid-state dewetting of quad-layer Ag/Au/Pd/Pt thin films on c-plane sapphire
Source: PLoS One. 2019 Oct 21;14(10):e0224208. doi: 10.1371/journal.pone.0224208 (PMC6802835; doi:10.1371/journal.pone.0224208)
Supplement: S7 Fig — (DOCX) [file pone.0224208.s007.docx]

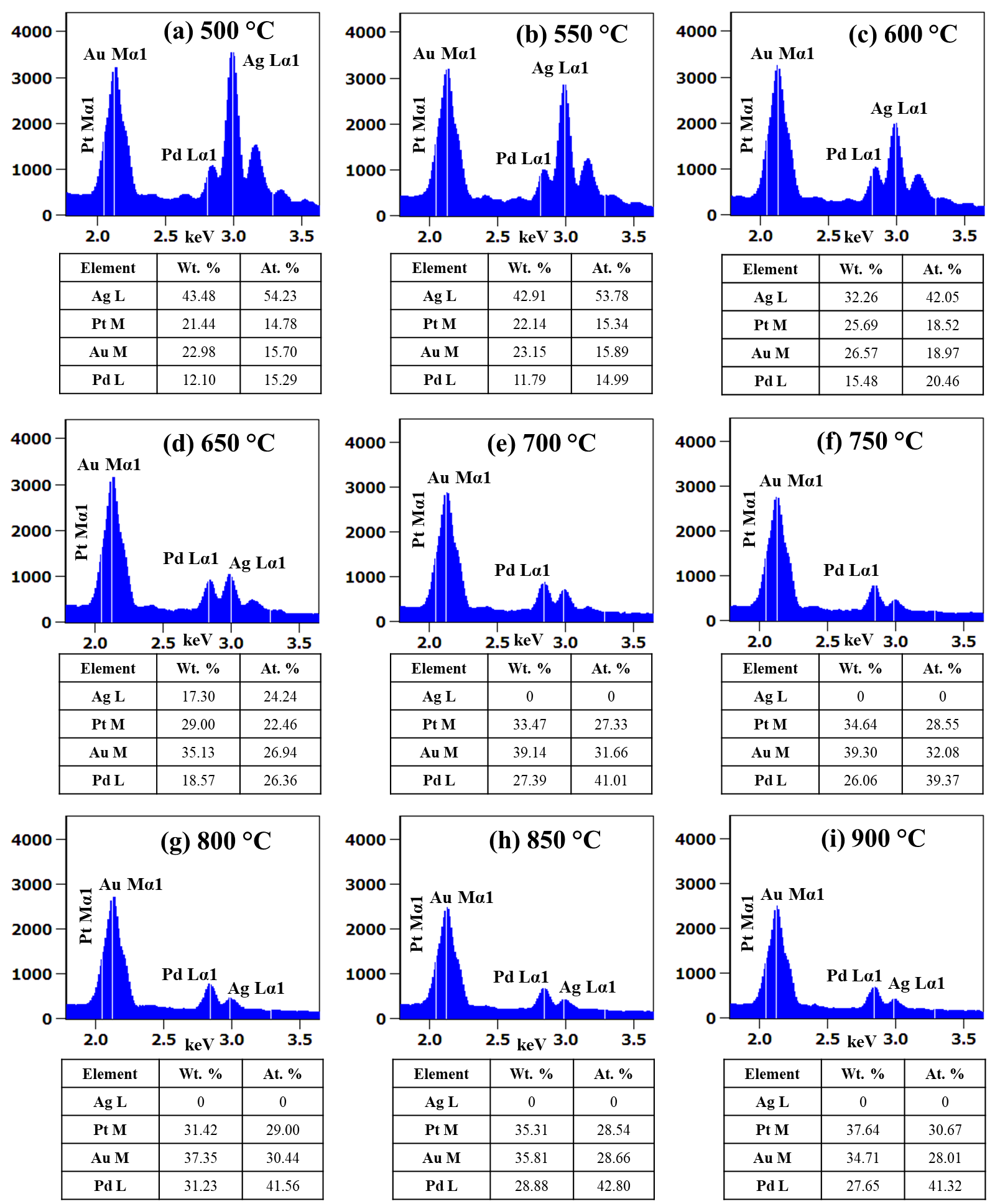


**Figure S7:** EDS spectral analysis of various alloy nanostructure fabricated with the Ag_24 nm_ / Au_9 nm_ / Pd_9 nm_ / Pt_9 nm_ quad-layer films at various annealing temperatures as labeled.
