# Supplementary material for: Systematic investigation on quad-metallic AgAuPdPt and tri-metallic AuPdPt NPs through the solid-state dewetting of quad-layer Ag/Au/Pd/Pt thin films on c-plane sapphire
Source: PLoS One. 2019 Oct 21;14(10):e0224208. doi: 10.1371/journal.pone.0224208 (PMC6802835; doi:10.1371/journal.pone.0224208)
Supplement: S8 Fig — (a) SEM image. (b) Enlarged SEM image. (c) EDS line profile across the nanocluster and void regions. (d)–(h) Elemental phase mapping of Ag, Au, Pd, Pt and Al. (DOCX) [file pone.0224208.s008.docx]

**
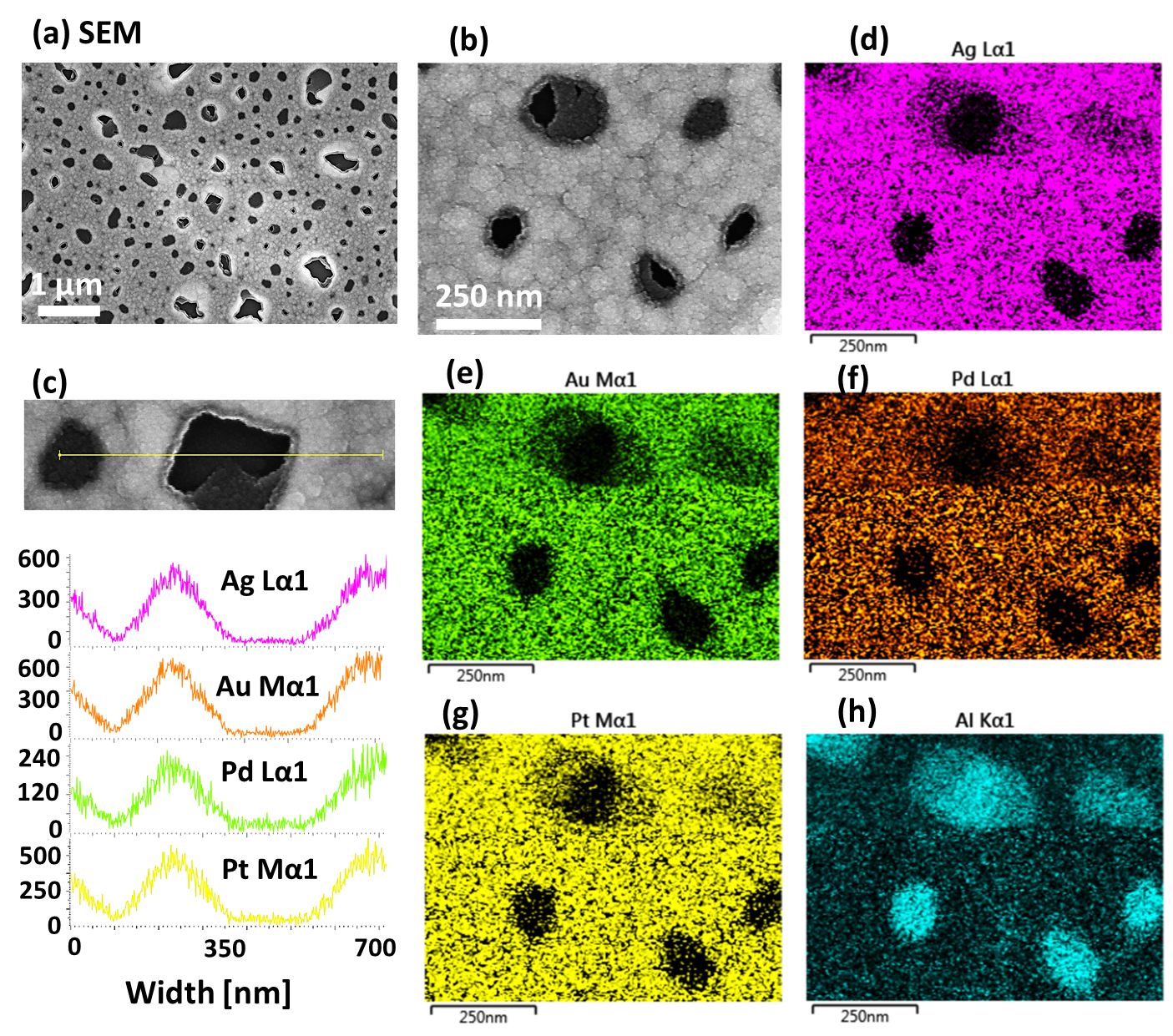
**

**Figure S8:** Elemental analysis of the AgAuPdPt with the Ag_24 nm_ / Au_9 nm_ / Pd_9 nm_ / Pt_9 nm_ quad-layer films by annealing between 550 ^o^C. (a) SEM image. (b) Enlarged SEM image. (c) EDS line profile across the nanocluster and void regions. (d) – (h) Elemental phase mapping of Ag, Au, Pd, Pt and Al.
