# Supplementary material for: Systematic investigation on quad-metallic AgAuPdPt and tri-metallic AuPdPt NPs through the solid-state dewetting of quad-layer Ag/Au/Pd/Pt thin films on c-plane sapphire
Source: PLoS One. 2019 Oct 21;14(10):e0224208. doi: 10.1371/journal.pone.0224208 (PMC6802835; doi:10.1371/journal.pone.0224208)
Supplement: S10 Fig — (a) AFM image. (b) Simulated extinction plot of the corresponding AuPdPt NP. (c) E-field profile in xy-plane at resonance wavelength. (d) E-field vector plots. (DOCX) [file pone.0224208.s010.docx]

**
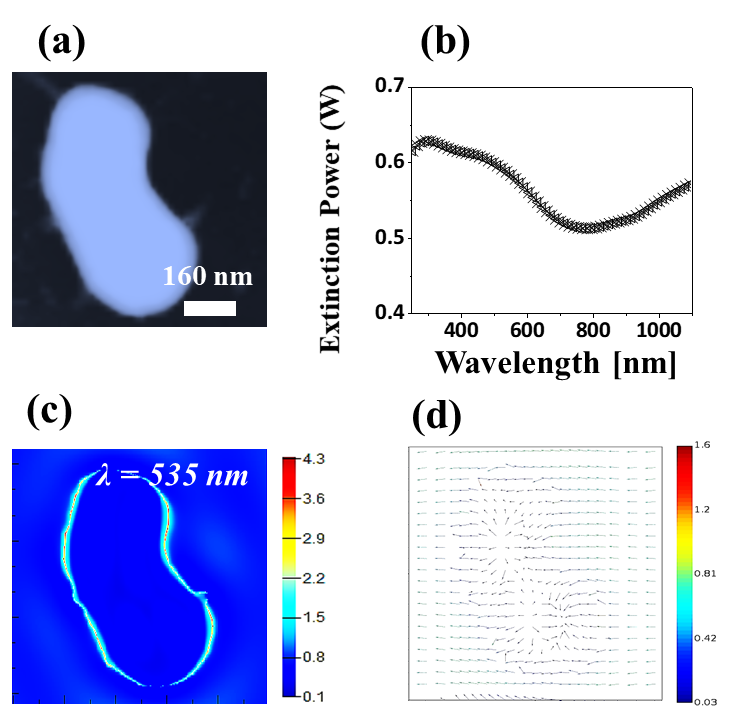
**

**Figure S10:** Finite difference time domain (FDTD) simulation of the typical AuPdPt alloy NP fabricated with the Ag_24 nm_ / Au_9 nm_ / Pd_9 nm_ / Pt_9 nm_ at 750 ^o^C for 120 s. (a) AFM image. (b) Simulated extinction plot of the corresponding AuPdPt NP. (c) E-field profile in xy-plane at resonance wavelength. (d) E-field vector plots.
