# Supplementary material for: Systematic investigation on quad-metallic AgAuPdPt and tri-metallic AuPdPt NPs through the solid-state dewetting of quad-layer Ag/Au/Pd/Pt thin films on c-plane sapphire
Source: PLoS One. 2019 Oct 21;14(10):e0224208. doi: 10.1371/journal.pone.0224208 (PMC6802835; doi:10.1371/journal.pone.0224208)
Supplement: S12 Fig — (a)–(i) AFM side-views of 5 × 5 μm2. (a-1)–(i-1) Corresponding cross-sectional line-profiles. (DOCX) [file pone.0224208.s012.docx]

**
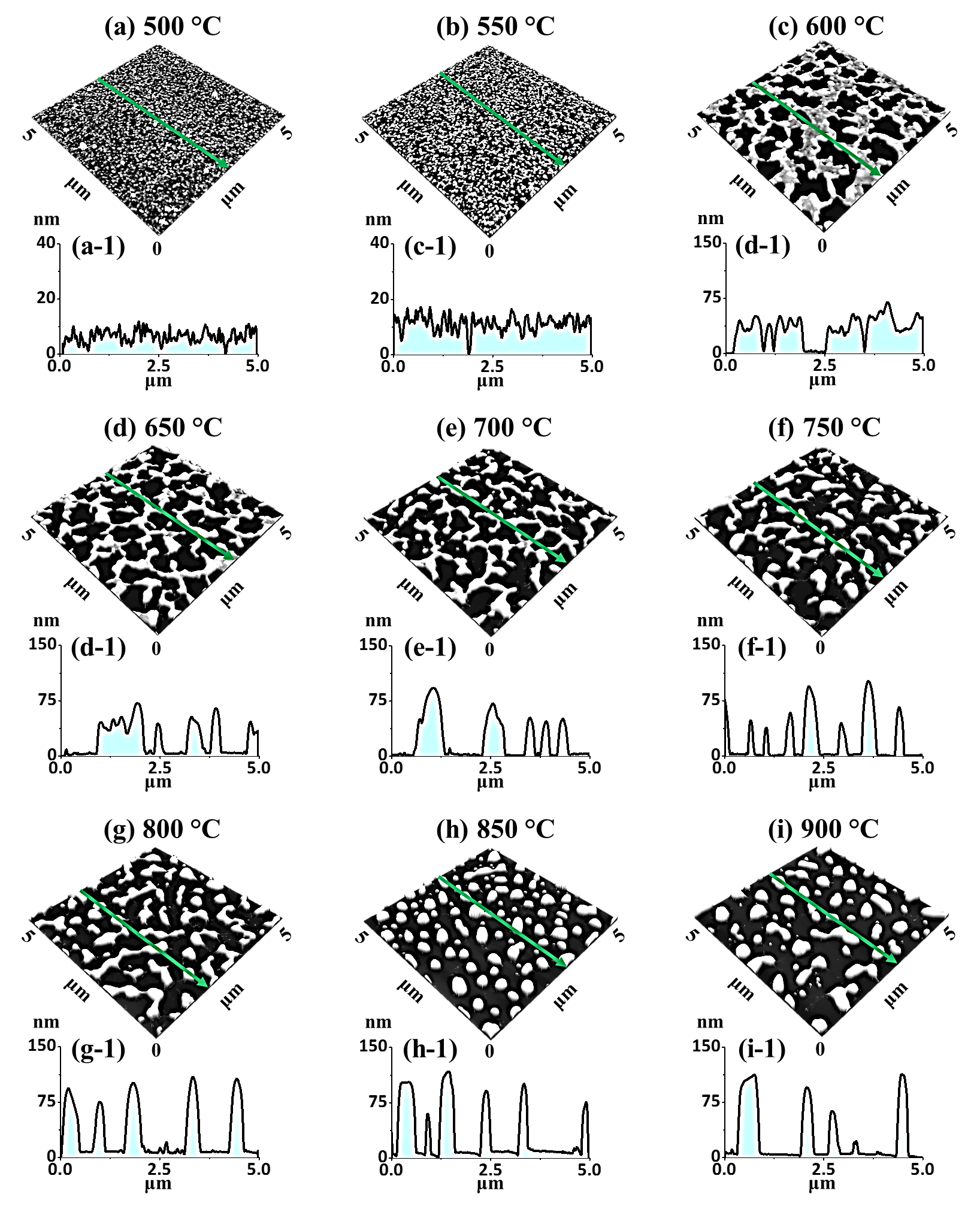
**

**S12 Fig.** Evolution of AuPdPt alloy nanostructures from connected to isolated NPs on saaphire (0001) by the systemetic control of annealing temperature between 500 and 900 ^o^C for 120 s with Ag_12 nm_ / Au_4.5 nm_ / Pd_4.5 nm_ / Pt_4.5 nm_ films. (a) – (i) AFM side-views of 5 × 5 µm^2^. (a-1) – (i-1) Corresponding cross-sectional line-profiles.
