# Supplementary material for: Systematic investigation on quad-metallic AgAuPdPt and tri-metallic AuPdPt NPs through the solid-state dewetting of quad-layer Ag/Au/Pd/Pt thin films on c-plane sapphire
Source: PLoS One. 2019 Oct 21;14(10):e0224208. doi: 10.1371/journal.pone.0224208 (PMC6802835; doi:10.1371/journal.pone.0224208)
Supplement: S13 Fig — (a)–(d) SEM images of corresponding alloy nanostructures between 600 and 900 °C. (a-1)–(d-1) AFM top-views of 3 × 3 μm2. (a-2)–(d-2) Magnified AFM side-views of 1 × 1 μm2. (a-3)–(d-3) Cross-sectional line-profiles. (e)–(g) Plots of average roughness (Ra), RMS roughness (Rq) and surface area ratio (SAR) of corresponding alloy nanostructures as a function of annealing temperature. (DOCX) [file pone.0224208.s013.docx]

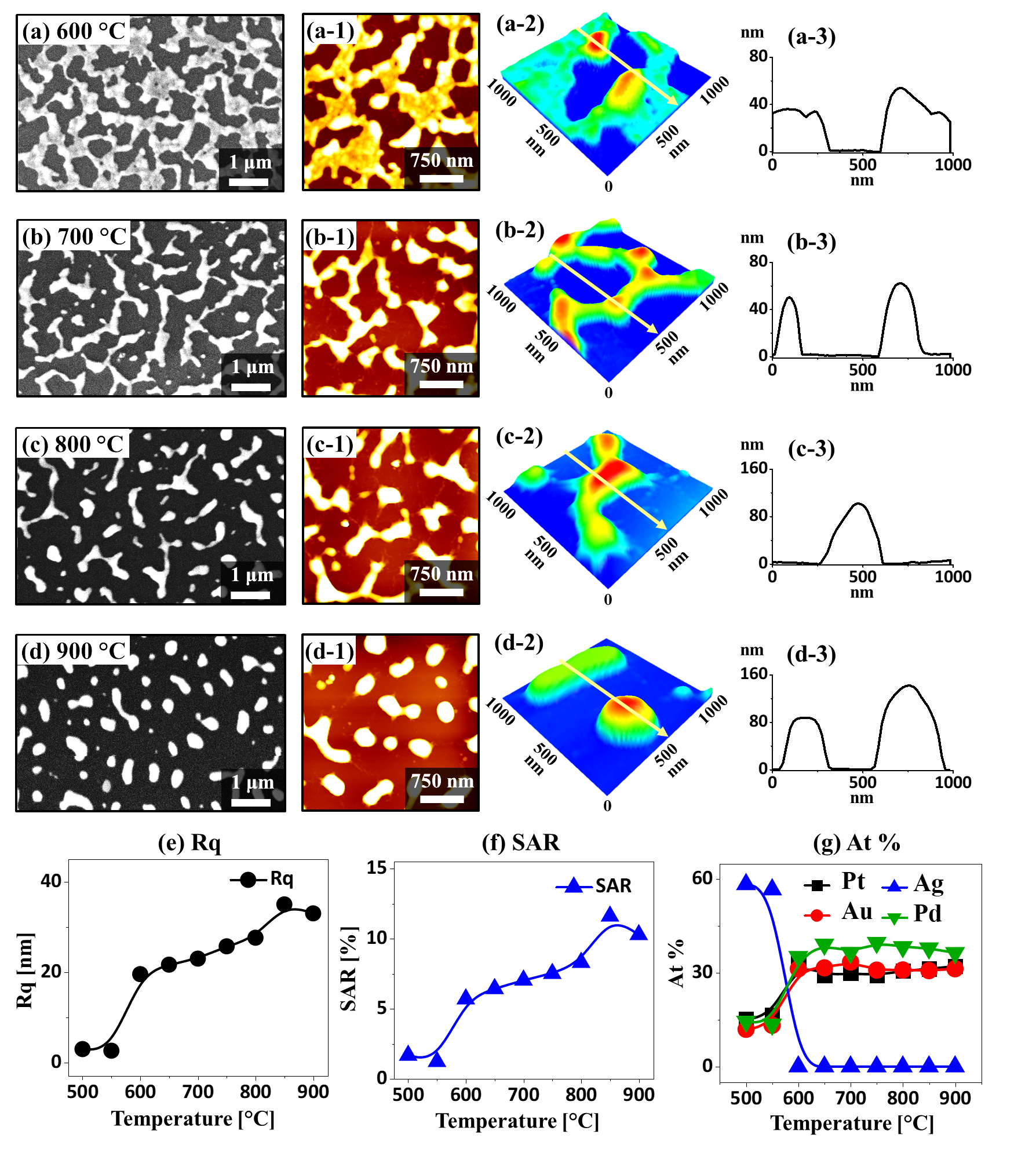


**S13 Fig.** Evolution of self-assembled alloy nanostructures fabricated with the Ag_12 nm_ / Au_4.5 nm_ / Pd_4.5 nm_ / Pt_4.5 nm_ quad-layers. (a) – (d) SEM images of corresponding alloy nanostructures between 600 and 900 ^o^C. (a-1) – (d-1) AFM top-views of 3 × 3 µm^2^. (a-2) – (d-2) Magnified AFM side-views of 1 × 1 µm^2^. (a-3) – (d-3) Cross-sectional line-profiles. (e) – (g) Plots of average roughness (Ra), RMS roughness (Rq) and surface area ratio (SAR) of corresponding alloy nanostructures as a function of annealing temperature.
