# Supplementary material for: Systematic investigation on quad-metallic AgAuPdPt and tri-metallic AuPdPt NPs through the solid-state dewetting of quad-layer Ag/Au/Pd/Pt thin films on c-plane sapphire
Source: PLoS One. 2019 Oct 21;14(10):e0224208. doi: 10.1371/journal.pone.0224208 (PMC6802835; doi:10.1371/journal.pone.0224208)
Supplement: S15 Fig — (DOCX) [file pone.0224208.s015.docx]

**
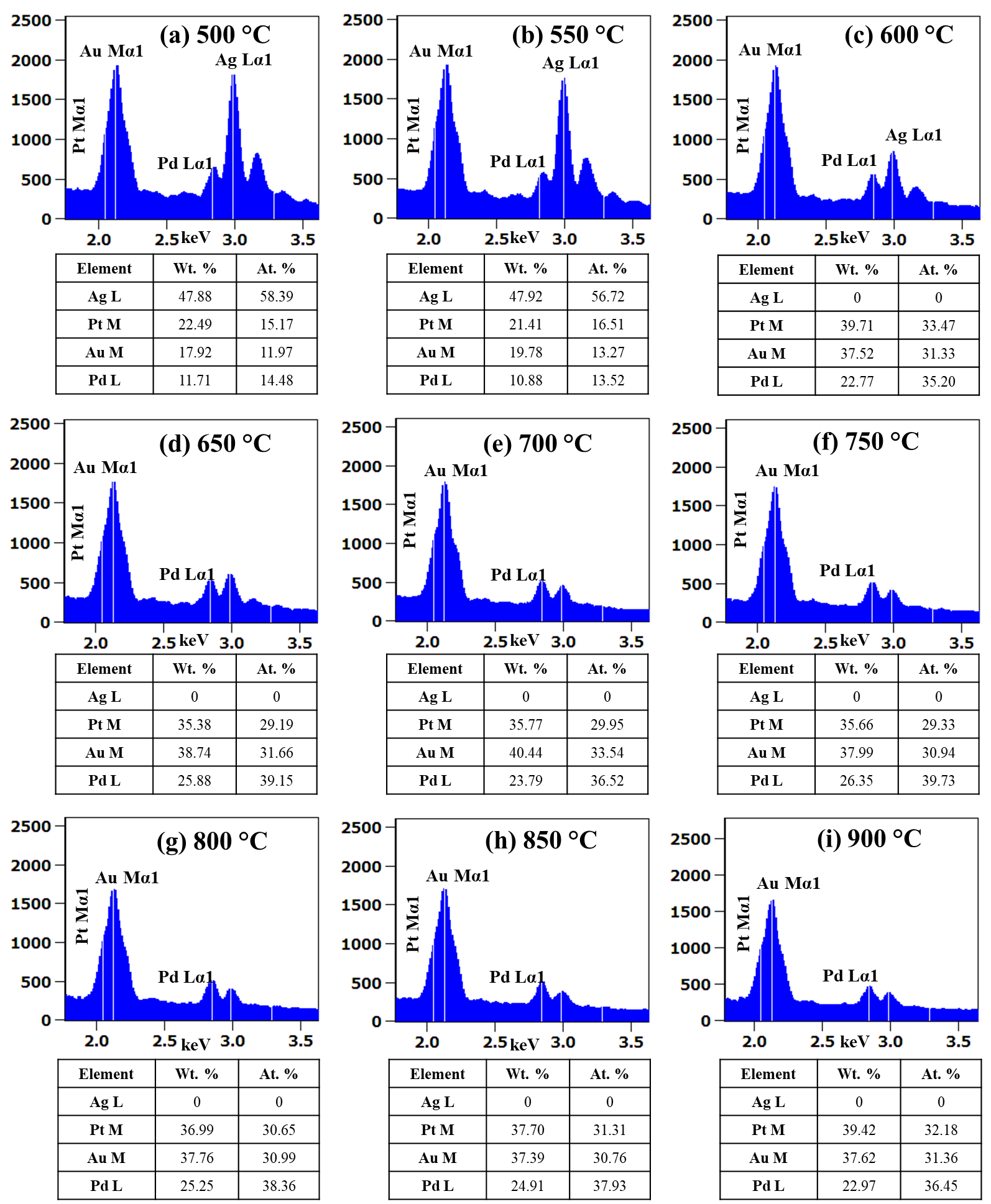
**

**S15 Fig.** EDS spectra of various alloy nanostructure fabricated with the Ag_12 nm_ / Au_4.5 nm_ / Pd_4.5 nm_ / Pt_4.5 nm_ films at various annealing temperatures as labeled.
