# Supplementary material for: Systematic investigation on quad-metallic AgAuPdPt and tri-metallic AuPdPt NPs through the solid-state dewetting of quad-layer Ag/Au/Pd/Pt thin films on c-plane sapphire
Source: PLoS One. 2019 Oct 21;14(10):e0224208. doi: 10.1371/journal.pone.0224208 (PMC6802835; doi:10.1371/journal.pone.0224208)
Supplement: S16 Fig — (a) Extinction spectra. (a-1)–(a-2) Normalized extinction spectra. (a-3) Magnified extinction spectra. (b) Reflectance spectra. (b-1)–(b-2) Normalized reflectance spectra. (b-3) Average reflectance. (c) Transmittance spectra. (c-1)–(c-2) Normalized transmittance spectra. (c-3) Average transmittance. (DOCX) [file pone.0224208.s016.docx]

**
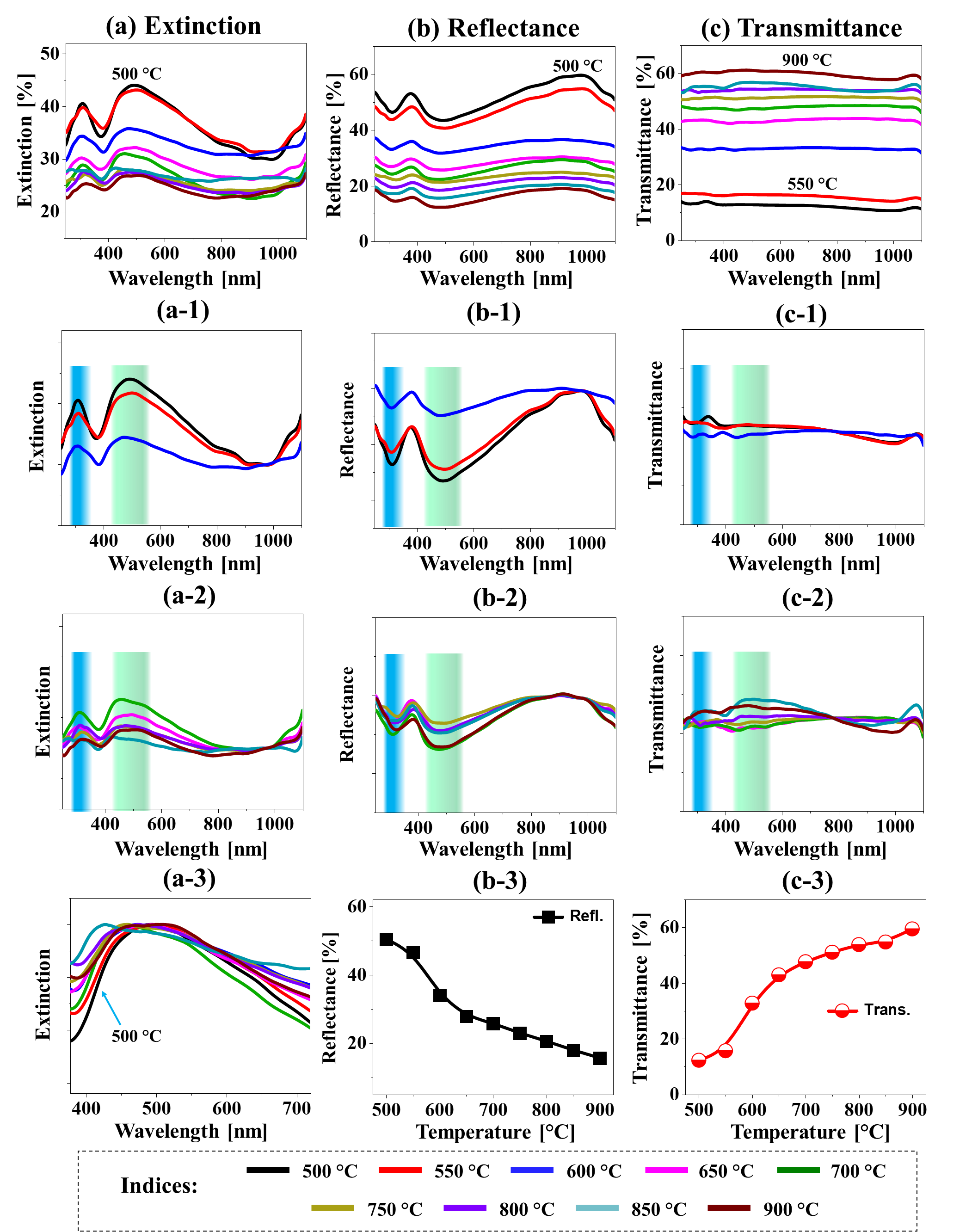
**

**S16 Fig.** Optical properties of alloy nanostructures fabricated with the Ag_12 nm_ / Au_4.5 nm_ / Pd_4.5 nm_ / Pt_4.5 nm_ quad-layers at various annealing temperature between 600 and 900 ^o^C. (a) Extinction spectra. (a-1) – (a-2) Normalized extinction spectra. (a-3) Magnified extinction spectra. (b) Reflectance spectra. (b-1) – (b-2) Normalized reflectance spectra. (b-3) Average reflectance. (c) Transmittance spectra. (c-1) – (c-2) Normalized transmittance spectra. (c-3) Average transmittance.
